# A Highly Sensitive Luminescent Biosensor for the Microvolumetric Detection of the *Pseudomonas aeruginosa* Siderophore Pyochelin

**DOI:** 10.1021/acssensors.1c01023

**Published:** 2021-09-03

**Authors:** Daniela Visaggio, Mattia Pirolo, Emanuela Frangipani, Massimiliano Lucidi, Raffaella Sorrentino, Emma Mitidieri, Francesca Ungaro, Andrea Luraghi, Francesco Peri, Paolo Visca

**Affiliations:** § Department of Science, Roma Tre University, Rome 00146, Italy; # Santa Lucia Fundation IRCCS, Rome 00179, Italy; ¶ Department of Biomolecular Sciences, University of Urbino Carlo Bo, Urbino 61029, Italy; ∥ Department of Molecular Medicine and Medical Biotechnology, University of Naples Federico II, Naples 80138, Italy; ⌉ Department of Pharmacy, University of Naples Federico II, Naples 80131, Italy; ● Department of Biotechnology and Biosciences, University of Milano-Bicocca, Milan 20126, Italy

**Keywords:** bioluminescence, biosensor, luciferase, pyochelin, *Pseudomonas aeruginosa*, siderophore

## Abstract

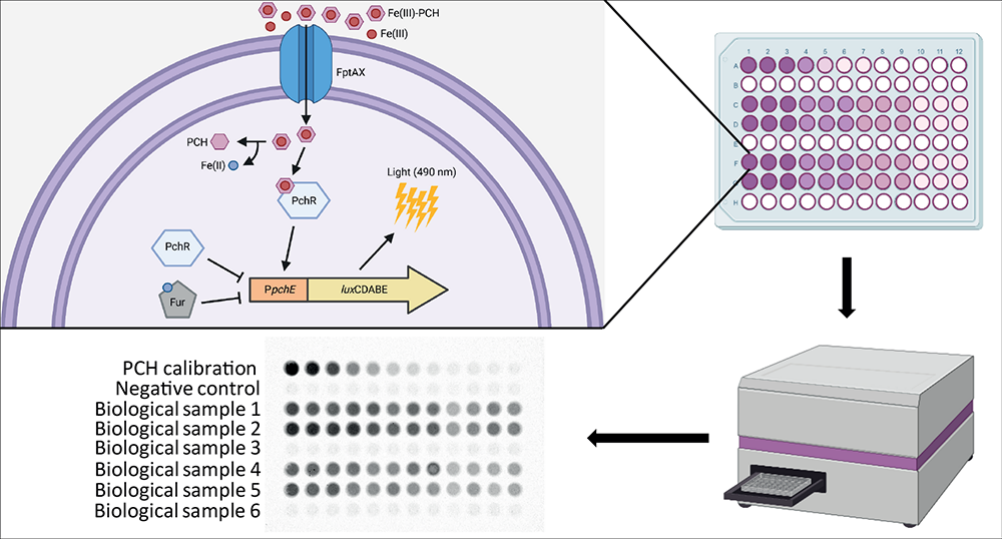

The pyochelin (PCH) siderophore produced by the pathogenic bacterium *Pseudomonas aeruginosa* is an important virulence factor, acting as a growth promoter during infection. While strong evidence exists for PCH production *in vivo*, PCH quantification in biological samples is problematic due to analytical complexity, requiring extraction from large volumes and time-consuming purification steps. Here, the construction of a bioluminescent whole cell-based biosensor, which allows rapid, sensitive, and single-step PCH quantification in biological samples, is reported. The biosensor was engineered by fusing the promoter of the PCH biosynthetic gene *pchE* to the *luxCDABE* operon, and the resulting construct was inserted into the chromosome of the Δ*pvdA*Δ*pchD*Δ*fpvA* siderophore-null *P. aeruginosa* mutant. A bioassay was setup in a 96-well microplate format, enabling the contemporary screening of several samples in a few hours. A linear response was observed for up to 40 nM PCH, with a lower detection limit of 1.64 ± 0.26 nM PCH. Different parameters were considered to calibrate the biosensor, and a detailed step-by-step operation protocol, including troubleshooting specific problems that can arise during sample preparation, was established to achieve rapid, sensitive, and specific PCH quantification in both *P. aeruginosa* culture supernatants and biological samples. The biosensor was implemented as a screening tool to detect PCH-producing *P. aeruginosa* strains on a solid medium.

Tissues and biological fluids of mammals are protected from invading pathogens by iron-binding proteins, which represent major components of the innate immunity.^1^ Therefore, during infection, bacteria are faced with a tremendous iron-starvation stress, which is inductive of siderophore (iron chelator) synthesis.^2−4^ The Gram-negative bacterium *Pseudomonas aeruginosa* is one of the most dreaded opportunistic pathogens in the hospital setting and represents the first cause of morbidity and mortality in cystic fibrosis (CF) patients.^5^*P. aeruginosa* produces two siderophores, namely, pyoverdine (PVD) and pyochelin (PCH), which are endowed with very different structural and functional properties and capable of chelating Fe(III) with different affinities.^6,7^

PVD is a dihydroxyquinoline-containing green fluorescent peptide showing very high affinity for Fe(III) (Kf = 10^32^ M^–1^).^8,9^ The role of PVD in *P. aeruginosa* pathogenicity has extensively been studied, since PVD is produced in large amounts and can easily be quantified both *in vitro* and *in vivo*, thanks to its strong fluorescence emission.^10,11^

PCH is the second siderophore produced by *P. aeruginosa*(12) and originates from the enzymatic condensation of salicylic acid with two cysteine molecules.^13,14^ The PCH structure contains three asymmetric carbons (C-4′, C-2″, C-4″) and exists as a mixture of the two interconvertible diastereomers PCH I (4′R, 2″R, 4″R) and PCH II (4′R, 2″S, 4″R).^15^ Its affinity for Fe(III) is 10^28^ M^–2^.^16^

The PCH biosynthetic genes are organized in two operons, *pchDCBA* and *pchEFGHI*.^17^ A third operon, *fptABCX*, encodes the FptA outer membrane receptor and the FptX inner membrane permease, which mediate active transport of PCH inside the *P. aeruginosa* cell.^18^ In the cytoplasm, the PCH-Fe(III) complex binds the PchR regulator, which induces the expression of the PCH biosynthesis and uptake operons.^19^ PchR is a protein belonging to the AraC/XylS family of transcriptional regulators, which act as repressors or activators of gene expression depending on the absence or presence of a specific effector molecule, respectively. In particular, genes implicated in PCH biosynthesis and uptake are repressed by PchR in the absence of cytoplasmic PCH, while they are induced when PchR is activated upon PCH-(FeIII) binding.^20,21^ Since an excess of cytoplasmic iron is toxic for bacterial cells, all *P. aeruginosa* iron acquisition systems are repressed under iron-replete conditions by the binding of the ferric uptake regulator protein (Fur) to the promoter regions of PVD and PCH biosynthesis and uptake genes.^14,22^ This highly specific regulatory circuitry ensures PCH production when (i) cells are iron starved (hence, Fur repression is relieved) and (ii) PCH is effective in feeding the cell with iron (hence, PchR binds cytoplasmic PCH-Fe(III), activates PCH biosynthesis, and transport genes).

PCH has a major impact on *P. aeruginosa* pathogenesis, as it contributes to overcoming the iron starvation response of the host during bacterial infection.^23^ PCH is crucial for feeding *P. aeruginosa* cells with iron and contributes to pathogenicity in a mouse model of lung infection.^24^ Indirect evidence of *in vivo* expression of PCH biosynthesis genes was inferred from transcriptional profiling of *P. aeruginosa* during septicemia, urinary tract, lung, and wound infections,^25−28^ and the PCH transport gene *fptA* was identified among the most highly expressed virulence genes *in vivo.*(29) However, *in vivo* detection of PCH is problematic, and PCH production during *P. aeruginosa* infection was occasionally documented in CF sputum samples, which showed significant induction of *pch* genes.^11,26^*In vitro* studies suggest that PCH is also responsible for secondary pathogenic effects on host tissues through the generation of hydroxyl radicals in combination with pyocyanin, which is an extracellular phenazine compound produced by *P. aeruginosa.*(30,31)

The paucity of information about PCH production levels during *P. aeruginosa* infection is due to the complexity of available methods to quantify this siderophore in biological samples. At present, PCH quantification methods rely on solvent extraction followed by concentration of the extract and PCH purification and detection by thin-layer chromatography, spectrofluorimetry, or HPLC.^14,32−34^ These methods are time-consuming, require handling of hazardous solvents and specialized equipment for PCH extraction and quantification, respectively, and unavoidably cause non-negligible loss of material, which could ultimately result in the underestimation of the PCH concentration.

In this study, we report the construction of a bioluminescent whole cell-based biosensor for the rapid, specific, and single-step quantification of PCH, overcoming the numerous drawbacks of current PCH detection methods. The sensitivity, range of linear response, and specificity of the biosensor were experimentally determined. The biosensor was developed in the 96-well microtiter plate format and successfully used for PCH quantification in *P. aeruginosa* culture supernatants and biological samples, then adapted to the agar plate format for the qualitative screening of PCH-producing *P. aeruginosa* clinical isolates.

## Experimental Section

### Bacterial Strains and Growth Media

Bacterial strains and plasmids used in this study are listed in Table S1. *Escherichia coli* and *P. aeruginosa* were grown in Luria-Bertani broth (LB)^35^ and LB agar plates. The *P. aeruginosa* biosensor strain Δ*pvdA*Δ*pchD*Δ*fpvA* P*pchE::lux* is freely available to the scientific community and can be provided upon request to the corresponding authors. When required, antibiotics were used at the following concentrations: ampicillin (Ap, 100 μg/mL), tetracycline (Tc, 12.5 μg/mL), and gentamicin (Gm, 10 μg/mL) for *E. coli*; Gm (200 μg/mL), carbenicillin (Cb, 250 μg/mL), and Tc (100 μg/mL) for *P. aeruginosa. P. aeruginosa* strains were also grown in the iron-poor casamino acid (DCAA) medium^36^ and in casamino acid (CAA) agarose plates (10 g/L CAA, Difco; 15 g/L Certified Molecular Biology Agarose, Bio-Rad).

### PVD Extraction and Quantification

PVD produced by *P. aeruginosa* strains was quantified after 24 h growth at 37 °C in DCAA by measuring the absorbance at 405 nm of culture supernatants appropriately diluted in 0.1 M Tris–HCl, pH 8.0. Values were normalized to the cell density of the bacterial culture (OD_600_), as previously described.^37^ PVD was purified as previously described.^38^ Briefly, *P. aeruginosa* Δ*pchD* was grown in DCAA for 24 h. The culture supernatant was purified by filtration through a Sep-Pak C18 Vac-Cartridge 3 cc (Waters). The filtered culture supernatant containing PVD was loaded and washed with double-distilled water to remove unwanted components. PVD was then eluted with 50% (vol/vol) methanol, evaporated to dryness in a desiccator, and dissolved in a small volume of double distilled water. The PVD concentration was determined by spectrophotometric measurement of the apo form at OD_405_ (ε = 1.4 × 10^4^ M^–1^ cm^–1^).

### PCH Extraction and Quantification

PCH was extracted from *P. aeruginosa* Δ*pvdA* by the ethyl acetate extraction of acidified 36 h-old culture supernatants in DCAA. Briefly, the supernatant was adjusted to pH 1.5–2.0 with 1 N HCl and extracted with 1 volume of ethyl acetate.^39^ After evaporation of the organic phase, the dry residue was suspended in 100 μL of methanol. PCH extracts were purified by an automatized reverse-phase chromatographer equipped with a UV–vis detector (BIOTAGE Isolera One Flash Chromatography System, RP-C18 column, gradient water/methanol). The amount of apo-PCH was determined by spectrophotometric measurement at OD_330_ (ε = 4400 M^–1^ cm^–1^).^40^ A 40 mM of stock solution of PCH was prepared in dimethyl sulfoxide (DMSO) and stored at −20 °C until used. The ethyl acetate extract of PAO1, Δ*pvdA*, Δ*pchD*, and *P. aeruginosa* TR1 was also applied to a silica gel G (60F254) thin-layer chromatography (TLC) plate (Merck) using acetone:methanol:0.2 M acetic acid (5:2:1) as the mobile phase.^41^ PCH from TLC plates was qualitatively characterized by (i) yellow-green fluorescence emission under UV light^42^ and (ii) iron-binding capacity when sprayed with 0.1 M FeCl_3_ in 0.1 M HCl resulting in red-brown spots.^42^

### Chemical Synthesis of PCH and Enantio-PCH

The two enantiomers *N*-methyl-l-cysteine and *N*-methyl-d-cysteine, which are required in the final step of PCH and enantio-PCH chemical synthesis, were prepared according to literature procedures,^43,44^ starting from commercially available l- or d-cysteine. In this synthetic procedure (Figure S1), cysteine enantiomers were separately reacted with trityl alcohol in TFA to protect the thiol group as *S*-trytil, and then the amine group was protected as *tert*-butoxycarbonyl (Boc) carbamate that, after *N*-methylation with methyl iodide, gave the fully protected *S*-trityl, *N*-Boc, *N*-methyl l- or d-cysteine. The simultaneous deprotection of amino and thiol groups (Boc and trityl cleavage, respectively) gave the final *N*-methyl-l- or d-cysteine. PCH and enantio-PCH were then synthetized using the literature protocol^45^ over four steps (Figure S2). Commercially available 2-hydroxybenzonitrile was condensed with l-cysteine providing the thiazolidine intermediate. The treatment of the thiazolidine intermediate with *N*-methyl-*O*-methyl hydroxylamine in the presence of condensing agents afforded the corresponding Weinreb amide, and the subsequent reduction with lithium aluminum hydride (LAH) gave aldehyde as a racemic mixture. The final condensation of aldehyde with the previously synthetized *N*-methyl-l-cysteine or its enantiomer *N*-methyl-l-cysteine followed by spontaneous cyclization gave PCH and enantio-PCH, respectively, as a mixture of four diastereomers (Figure S2).

### Generation of Plasmids and Reporter Strains

Unmarked in-frame deletion mutants in *fpvA* and *pchR* genes were constructed by suicide plasmid insertion mutagenesis. *E. coli* was used for recombinant DNA manipulations. The constructs for mutagenesis were generated by directional cloning into the pDM4 or pME3087 vector (Table S1)^46,47^ of two DNA fragments of ∼600 bp, encompassing the regions upstream and downstream of the sequence to be deleted. Fragments were amplified by PCR, digested with the appropriate restriction enzymes, and cloned into pDM4 or pME3087, generating the derivative vectors pDM4Δ*fpvA* and pME3087Δ*pchR* (Table S1). PCR primers and restriction enzymes used for cloning of PCR products are listed in Table S2. All constructs were verified by DNA sequencing. Deletion vectors were conjugally transferred from *E. coli* S17.1λ*pir* into the *P. aeruginosa* suitable deletion mutants (Table S1). The in-frame deletion mutations were obtained by recombination, as previously described.^46,47^ All the deletion events were verified by PCR using primers flanking the deleted region and amplicon sequencing. For the generation of mini-CTX P*pchE::lux* insertion, a DNA fragment encompassing the *pchE* promoter was amplified using the primers listed in Table S2 from the *P. aeruginosa* PAO1 chromosome. The amplicon was digested with BamHI-HindIII and ligated in the mini-CTX-lux plasmid. The resulting mini-CTX P*pchE::lux* was transferred from *E. coli* S17.1λ*pir* into *P. aeruginosa* strains by conjugation. Excision of the mini-CTX plasmid was achieved by Flp-mediated recombination *via* pFLP2, as previously described.^48^

### Biosensor Response to PCH and Other Iron-Binding Compounds

To test the biosensor response to PCH, *P. aeruginosa* PAO1 Δ*pvdA*Δ*pchD*Δ*fpvA* P*pchE::lux* was grown for 16 h at 37 °C in DCAA supplemented with 1 μM FeCl_3_. To exclude any iron carryover, the cells were washed with saline prior to being suspended in DCAA. The biosensor strain PAO1 Δ*pvdA*Δ*pchD*Δ*fpvA* P*pchE::lux* was inoculated at final OD_600_ of 1, 0.5, and 0.25 into black, clear-bottom 96-wells microtiter plates (Greiner) in the presence of an increasing concentration of purified PCH. Different inoculum volumes (200, 100, and 50 μL) were tested in the assay. OD_600_ and light count per second (LCPS) were monitored every 15 min in a Tecan Spark 10 M multilabel plate reader (Tecan, Männedorf, Switzerland) for up to 6 h at 25 °C. Once the optimal biosensor assay condition is defined (OD_600_ = 0.25; final volume 50 μL per well), these parameters were applied in all subsequent assays. The limit of detection (LOD) and the limit of quantification (LOQ) of the PCH biosensor were determined according to the equations: LOD = 3 × (SD/*S*) and LOQ = 10 × (SD/*S*),^49,50^ where SD is the standard deviation of the blank value (*n* = 10 replicates) and *S* is the slope of the calibration curve (*i.e.*, the sensitivity). The biosensor response was also assessed in the presence of chemically synthesized PCH and enantio-PCH, purified PVD, sodium salicylate (Sigma), deferoxamine (Desferal; Novartis), deferiprone (3-hydroxy-1,2-dimethylpyridin-4(1*H*)-one; Sigma-Aldrich), and FeCl_3_ (Sigma) at the indicated concentrations.

### Biological Fluids and CF Sputa

Sputum and first-morning urine specimens were collected from 10 healthy donors who tested culture-negative for *P. aeruginosa.* Each fluid was pooled, centrifuged (13,000*g*, 10 min, 4 °C), and sterilized through a 0.20 μm filter. Sterile artificial tears (Irilens 0.4%) were purchased from Montefarmaco (Italy). In addition, sputa from eight anonymous CF patients were provided by the clinical microbiology laboratory of Policlinico Umberto I, Rome (Italy), and processed as described above. Biological samples were stored at −20 °C until the assay.

### PCH Quantification in *P. aeruginosa* Culture Supernatants and Biological Fluids

PCH detection in culture supernatants of *P. aeruginosa* strains and biological fluids (including CF sputa) was performed using the *P. aeruginosa* Δ*pvdA*Δ*pchD*Δ*fpvA* P*pchE::lux* biosensor. *P. aeruginosa* strains (listed in Table S1) were grown for 24 h at 37 °C in DCAA. The culture supernatants were collected, filtered through a Millipore membrane (pore size 0.45 μm, Sarstedt), and stored at −20 °C until used. Five microliters of appropriate dilutions of culture supernatants or biological fluids were added to 45 μL of DCAA inoculated with the biosensor strain (final OD_600_ = 0.25). Microtiter plates were incubated at 25 °C, and OD_600_ and LCPS were measured after 3.5 h using a Tecan Spark 10 M multilabel plate reader. A calibration curve was generated with purified PCH at known concentrations (5–320 nM) and used to calculate the concentration of PCH in each sample.

### Detection of PCH and Siderophores on Agar Plates

Single colonies of different *P. aeruginosa* strains (listed in Table S1) were cultured for 8 h in LB at 37 °C. Bacteria were washed in saline, diluted to OD_600_ = 0.1, and 5 μL of each bacterial suspension was spotted onto casamino acid (CAA) agarose plate (5 g/L CAA and 15 g/L agarose). After 24 h incubation at 37 °C, PVD production was detected under UV light, and cells were killed through exposure to chloroform vapors for 15 min. Plates were then subsequently overlaid with CAA soft agarose (5 g/L casamino acids, Difco; 7.5 g/L agarose Bio-Rad) containing the biosensor strain (OD_600_ = 0.5). After incubation at 37 °C for 1 h, plates were visualized with a ChemiDoc XRS+ Imaging System (Bio-Rad), using a 3 min exposure time.

## Results and Discussion

### Design and Construction of a PCH-Responsive Whole Cell-Based Biosensor

**Figure 1:**
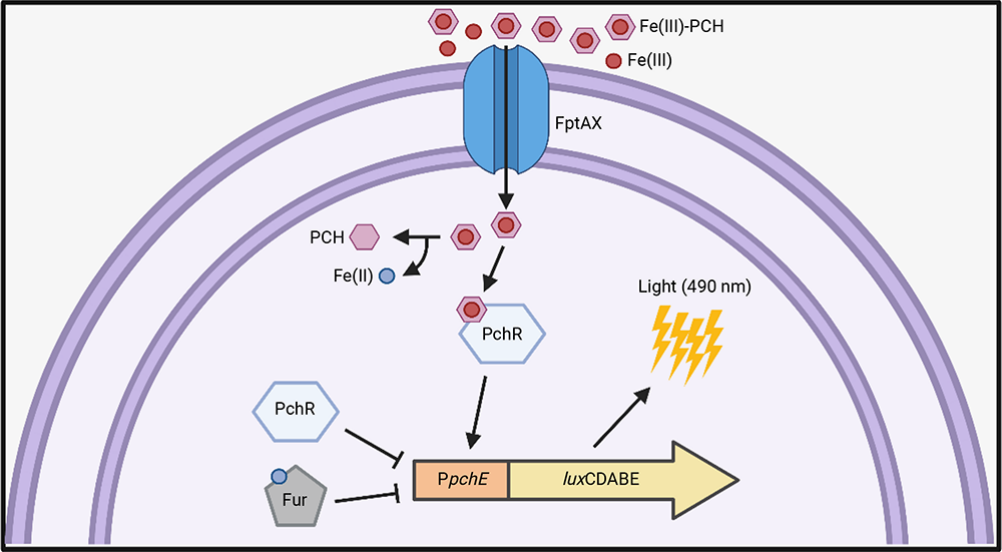
Schematic of the PCH-responsive whole cell-based biosensor. A transcriptional fusion between the *pchE* promoter (P*pchE*) and the *luxCDABE* operon was integrated in the chromosome of the *P. aeruginosa* Δ*pvdA*Δ*pchD*Δ*fpvA* mutant. Fe(III)-PCH is actively transported in the cytoplasm via FptAX, where it binds the transcriptional regulator PchR and directs the expression of the P*pchE::lux* fusion, eliciting bioluminescence emission. The Fe(II)-bound ferric uptake regulator Fur and PCH-unloaded PchR repress the expression of the P*pchE*::*lux* fusion. T-shaped lines represent negative control of P*pchE*.

To generate a biosensor capable of quantifying PCH at the nanomolar level, the *luxCDABE* operon, encoding the luciferase enzyme from the bacterium *Photorhabdus luminescens*, was chosen as the reporter system, being characterized by a high signal/noise ratio and not requiring supplementation with an exogenous substrate for signal (blue photon) emission.^51,52^ A transcriptional fusion between the PchR-dependent P*pchE* promoter and the *luxCDABE* operon was generated and integrated in the *attB* neutral chromosomal site in the *P. aeruginosa* Δ*pvdA*Δ*pchD*Δ*fpvA* triple mutant^53^ (Figure 1). This recipient strain is a siderophore null mutant, *i.e.*, impaired in the synthesis of both PVD and PCH, and is also unable to uptake PVD due to the absence of the FpvA receptor. The inability to produce siderophores prevents P*pchE* auto-induction by endogenously synthesized PCH. Moreover, the lack of the PVD transporter FpvA prevents the entrance of exogenous PVD into the biosensor cell, thus avoiding the repression of the P*pchE::lux* fusion by Fe(III) delivered through the Fe(III)-PVD uptake pathway. However, the Δ*pvdA*Δ*pchD*Δ*fpvA* mutant still produces both FptA and FptX transporters, so it has no defect in PCH uptake from the extracellular milieu. Consequently, we predicted (i) the bioluminescence emission of the whole-cell biosensor to be dependent on the levels of exogenously added PCH, which is transported intracellularly and serves as inducer of the P*pchE*::*luxCDABE* promoter-probe gene fusion, and (ii) the bioluminescence emission to be proportional to the PCH concentration (Figure 1).

### Experimental Setup for the Use of the Bioluminescent Whole Cell-Based Biosensor

The expression of *pch* (PCH biosynthesis) genes and, consequently, of the P*pchE::luxCDABE* reporter fusion is repressed by Fur under iron replete conditions. This is because the P*pchE* promoter contains a Fur-Fe(II)-binding region, which abrogates the transcriptional activity under conditions of iron availability.^22^

In order to prevent Fur-Fe(II)-mediated repression of the biosensor, the iron-poor medium DCAA^36^ was chosen for setting the operational conditions of the bioluminescent biosensor. Although iron scarcity is essential for studying the biosensor response to PCH, too severe iron limitation imposed by DCAA (<0.5 μM)^36^ would suppress the growth of a siderophore-null *P. aeruginosa* mutant. Therefore, the minimum non-limiting iron concentration allowing the Δ*pvdA*Δ*pchD*Δ*fpvA* mutant to grow similarly to the wild type, without causing complete repression of iron uptake genes, was initially investigated. To this purpose, the growth of the biosensor strain and wild-type PAO1 was monitored for 24 h in DCAA supplemented with increasing FeCl_3_ concentrations (from 0.5 to 8 μM), and PVD production by wild-type PAO1 was measured as an indicator of iron-repressible gene expression. *P. aeruginosa* PAO1 grew poorly in the absence of exogenously added iron, and the Δ*pvdA*Δ*pchD*Δ*fpvA* mutant grew even less (Figure S3A,B). However, the addition of 1 μM FeCl_3_ supported the sufficient growth of both strains without abrogating PVD production by wild-type PAO1 (Figure S3C). Therefore, biosensor cells were cultivated for 24 h in DCAA supplemented with 1 μM FeCl_3_, since this iron concentration does not shut off the expression of Fur-Fe(II)-repressible genes (*i.e.*, *pvd* and *pch* genes).

**Figure 2:**
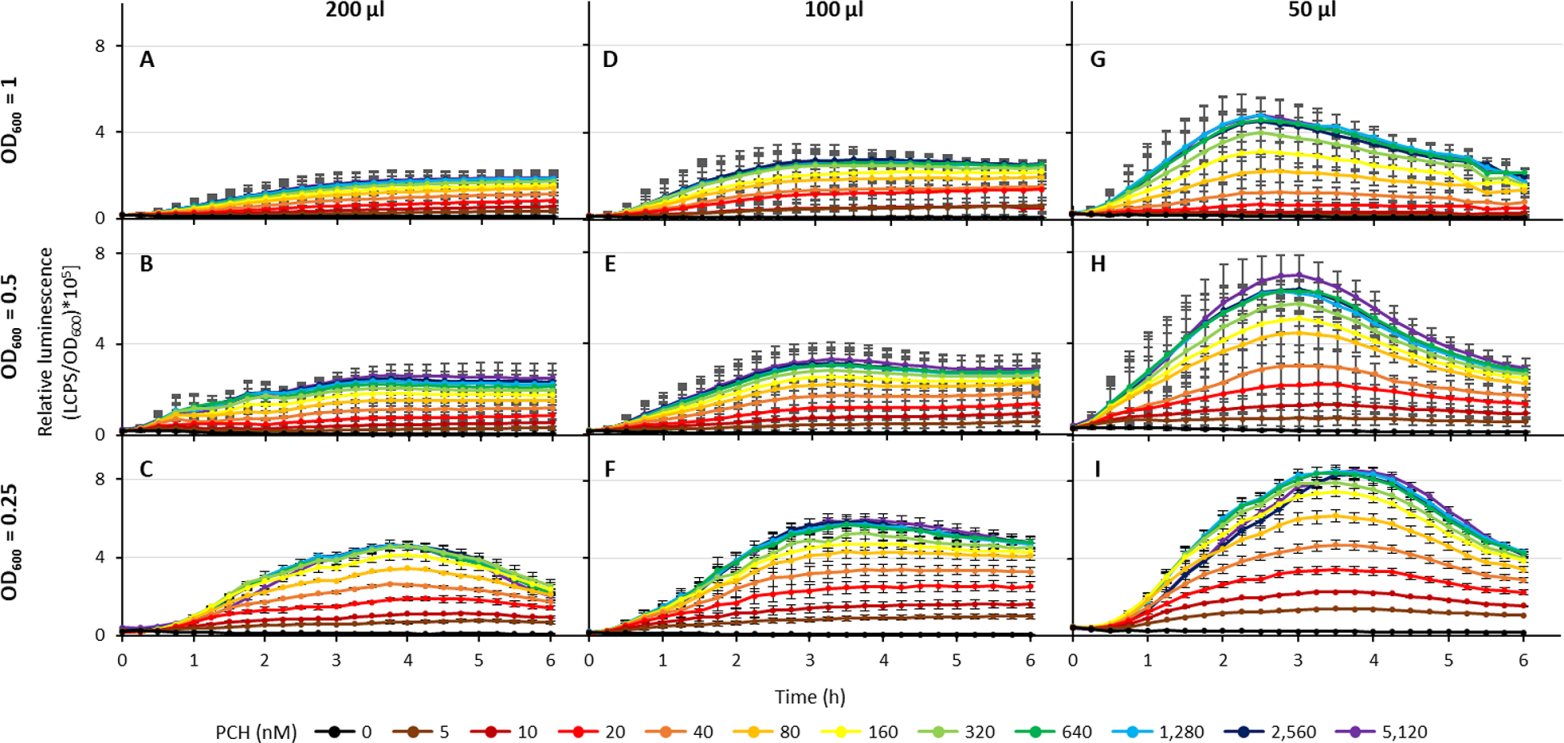
Effect of reporter cell density and assay volume on the biosensor response to PCH. Relative light emission (LCPS/OD_600_) by the *P. aeruginosa* Δ*pvdA*Δ*pchD*Δ*fpvA* mutant carrying the P*pchE::lux* fusion, in response to increasing PCH concentrations, ranging from 5 to 5120 nM. LCPS and OD_600_ were measured every 15 min for 6 h at 25 °C. Bacteria were inoculated at three different cell densities and three final volume combinations: (A) 200 μL, OD_600_ = 1; (B) 200 μL, OD_600_ = 0.5; (C) 200 μL, OD_600_ = 0.25; (D) 100 μL, OD_600_ = 1; (E) 100 μL, OD_600_ = 0.5; (F) 100 μL, OD_600_ = 0.25; (G) 50 μL, OD_600_ = 1; (H) 50 μL, OD_600_ = 0.5; (I) 50 μL, OD_600_ = 0.25. Data are the mean of three independent experiments ± SD.

To set the optimal experimental conditions for luminescence detection, three cell densities (OD_600_ = 1.0; 0.5; 0.25) and three different volumes of the bacterial suspension (200, 100, and 50 μL) were tested in a 96-well microtiter plate assay. All the experiments were performed in DCAA supplemented with purified PCH from *P. aeruginosa* PAO1 (concentration range 5 to 5120 nM), and the relative bioluminescence emission was monitored every 15 min for 6 h (Figure 2). To minimize the growth of the biosensor strain, the temperature was set at 25 °C during the assay. Interestingly, the lowest cell density (OD_600_ ≃ 0.25) and the smallest volume (50 μL) provided the highest relative light emission, expressed as light counts per second (LCPS)/OD_600_ of the cell suspension (Figure 2I), indicating that the assay volume influences the amount of light detectable per cell and hence the biosensor response. This result can be explained by the multiple scattering effect that occurs when the concentration of particles (*i.e.*, cells) is too high. In this case, the measured OD_600_ does not linearly increase with the cell number, and the Beer–Lambert law is no longer a valid approximation. In addition, the bioluminescent photons are scattered by overlying cells and are deflected away from the photodetector.^54^ This is because bacteria located in the upper layers shield the light signal emitted by the bacteria underneath. In fact, the higher the volume, the higher the optical path of the well, hence the light shielding by the bacterial suspension. Lowering the reaction volumes and cell densities enhanced the relative performance of the biosensor, expressed as LCPS/OD_600_. An optimal dose-dependent response of the biosensor was observed using the lowest cell density (OD_600_ ≃ 0.25) and the smallest assay volume (50 μL) within the 5–160 nM PCH concentration range. PCH concentrations of >160 nM caused a moderate increase in light emission (Figure 2I). This phenomenon is likely due to the fact that PCH concentrations higher than 160 nM saturate the FptA/FptX transporters and/or the transcriptional regulator PchR. Under these conditions, the Δ*pvdA*Δ*pchD*Δ*fpvA* P*pchE::lux* biosensor rapidly responded to exogenous PCH, attaining the maximum relative light emission in 3 to 3.5 h after the addition of PCH (Figure 2I). No significant evaporation of the biosensor suspension medium (*i.e.*, volume reduction) was noticed during the experimental time course.

### Bioluminescent Cell-Based Biosensor Selectively Responds to PCH, Requires PchR, and Is Repressed by Iron

**Figure 3:**
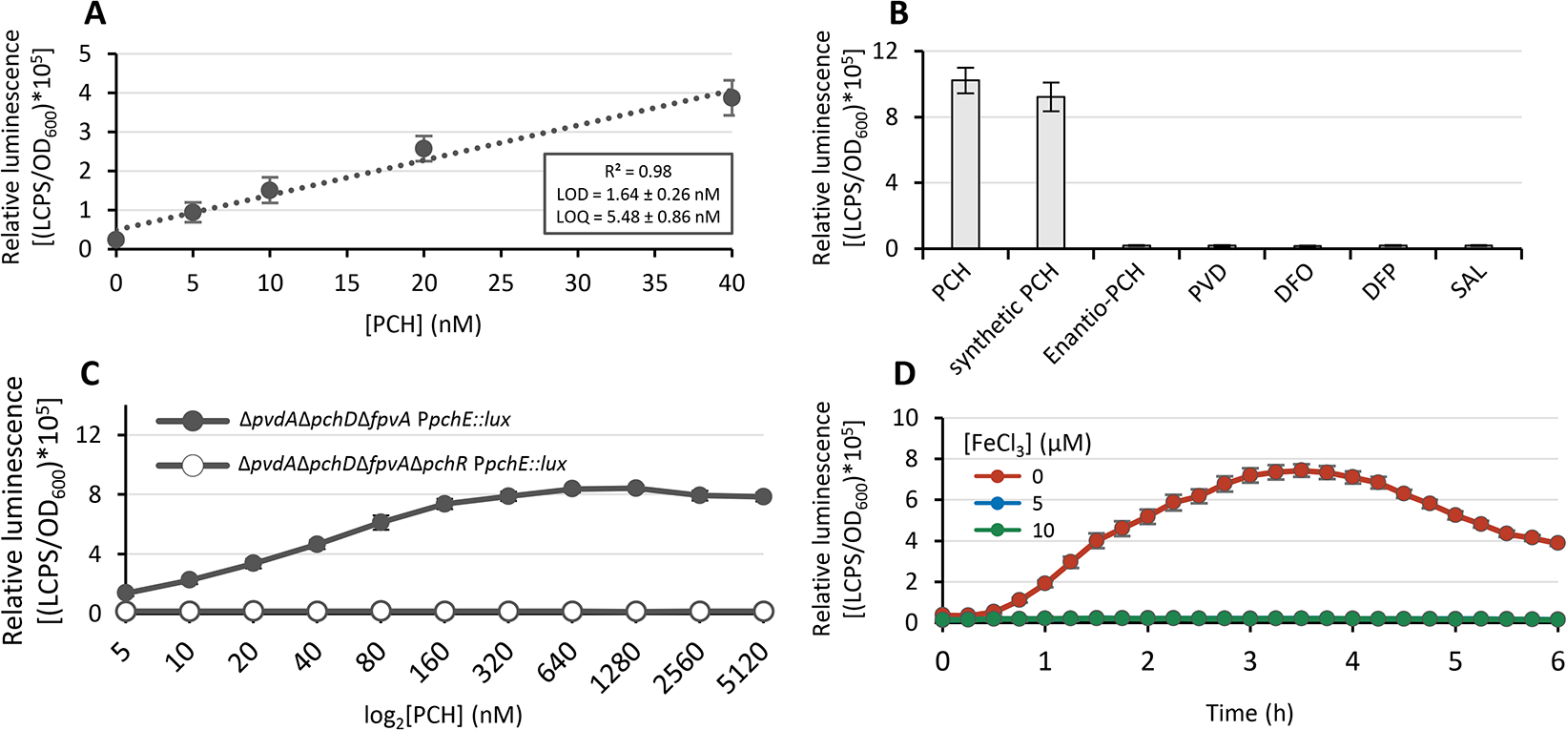
Biosensor response is activated by PchR and is repressed by iron. (A) Linear dose–response plot of the biosensor. Relative light emission values (LCPS/OD_600_) were taken after 3.5 h of incubation at 25 °C in the presence of increasing PCH concentration (0–40 nM). The linear regression line and the *R*^2^ value are shown. The limit of detection (LOD) and limit of quantification (LOQ) were calculated with S/N (signal/noise ratio) values of 3 and 10, respectively. (B) Response of the biosensor to natural PCH from *P. aeruginosa* PAO1, synthetic PCH, enantio-PCH, pyoverdine (PVD), deferoxamine (DFO) deferiprone (DFP), and salicylate (SAL) after 3.5 h of incubation at 25 °C. All chelators were added at the final concentration of 160 nM. (C) Relative light emission (LCPS/OD_600_) by the *P. aeruginosa* Δ*pvdA*Δ*pchD*Δ*fpvA* mutant carrying the P*pchE::lux* fusion (black circle) and by the *P. aeruginosa* Δ*pvdA*Δ*pchD*Δ*fpvA*Δ*pchR* mutant carrying the P*pchE::lux* fusion (white circle) in response to increasing PCH concentrations (5 to 5120 nM) after 3.5 h of incubation at 25 °C. (D) Relative light emission (LCPS/OD_600_) by the *P. aeruginosa* Δ*pvdA*Δ*pchD*Δ*fpvA* mutant carrying the P*pchE::lux* fusion in response to 160 nM PCH in the presence of 5 μM (square), 10 μM (triangle), or without (circle) FeCl_3_. LCPS and OD_600_ were measured every 15 min for 6 h at 25 °C. Data are the mean of three independent experiments ± SD.

Since the maximum light emission of the Δ*pvdA*Δ*pchD*Δ*fpvA* P*pchE::lux* biosensor was observed 3.5 h after the addition of PCH, the limit of detection (LOD) and the limit of quantification (LOQ) were determined at this time point. A linear dose–response relationship (*R*^2^ = 0.98) in the 5–40 nM range of PCH concentration was observed (Figure 3A). Within this PCH concentration range, the LOD and LOQ values were 1.64 ± 0.26 and 5.48 ± 0.86 nM, respectively (Figure 3A). These analytical performances represent a significant advance over previous PCH quantification methods, which required large sample volumes (mL)^36,42,55^ and eventual concentration after solvent extraction^36,42^ and exhibited sensitivity in the μM PCH range.^11^ To investigate the selectivity of the Δ*pvdA*Δ*pchD*Δ*fpvA* P*pchE::lux* biosensor, the luminescence emission was measured upon the addition of several iron-chelating compounds, using the standard test conditions (OD_600_ ≃ 0.25; 50 μL volume; 3.5 h; 25 °C). Purified PCH from *P. aeruginosa* PAO1 and chemically synthetized PCH were included as controls. Each iron-chelating compound (listed in Table S3) was added at 160 nM. As expected, the bioluminescence emission induced by the chemically synthesized PCH was similar to that of the native PCH extracted from *P. aeruginosa* culture supernatants (Figure 3B). Conversely, in the presence of chemically synthesized enantio-PCH, a PCH diastereoisomer was produced by *Pseudomonas protegens*,^56^ and the bioluminescence emission was completely abrogated (Figure 3B)*.* The inability of enantio-PCH to induce the expression of PCH genes in *P. aeruginosa* and consequently the biosensor response confirms that enantio-PCH is not recognized by *P. aeruginosa* due to the high specificity of the PCH translocation machinery and of the PchR-mediated gene expression.^55^ No response was also observed with other iron-chelating compounds, namely, PVD, deferoxamine (DFO), deferiprone (DFP), and sodium salicylate (SAL, a PCH precursor), further confirming the biosensor selectivity (Figure 3B). The biosensor genetic circuitry is based on the assumption that the production of PCH is controlled by the cytoplasmic transcriptional regulator PchR, which activates the transcription of PCH biosynthesis and transport genes. To define the role of PchR on the biosensor response, the *pchR* gene was deleted from the Δ*pvdA*Δ*pchD*Δ*fpvA* P*pchE::lux* biosensor strain, and the expression of the P*pchE::lux* fusion was monitored using the standard assay conditions (OD_600_ ≃ 0.25; 50 μL volume; 3.5 h; 25 °C concentration range). As expected, the deletion of *pchR* completely abrogated PCH-inducible P*pchE::lux* expression in the 5–5.120 nM PCH range, confirming that PchR is strictly required in PCH gene expression and thus for the PCH-mediated response of the biosensor (Figure 3C).

To verify the ability of iron to repress the expression of the P*pchE::lux* fusion, the biosensor was exposed to 160 nM of PCH, in the presence of two different FeCl_3_ concentrations (*i.e.*, 5 and 10 μM). The bioluminescence signal emission was completely shut off with 5 μM FeCl_3_ (Figure 3D).

Taken together, these results indicate that the biosensor response depends on the concentration of PCH and iron, in line with the regulatory mechanisms, which control PCH production and uptake.

### PVD Interference with PCH Measurement

Several studies in animal models and humans showed that PCH biosynthesis and uptake genes are expressed during *P. aeruginosa* infection,^25−29^ though the presence of PCH could be detected fluorometrically in a few CF sputum samples.^11,26^ Fluorescence spectroscopy can detect PCH concentrations of >1 μM, and therefore, with this method, it is not possible to detect PCH in *P. aeruginosa* isolates, which produce lower PCH concentrations (*i.e.*, <1 μM).^11^ Moreover, PCH fluorescence is quenched upon Fe(III) binding,^57^ implying that the Fe(III)-PCH complex escapes fluorometric detection. Another commonly used procedure for PCH quantification entails solvent extraction and purification by HPLC.^39^ Both these methods are affected by significant analyte loss and unavoidably underestimate the actual concentration of PCH produced by *P. aeruginosa*.

**Figure 4:**
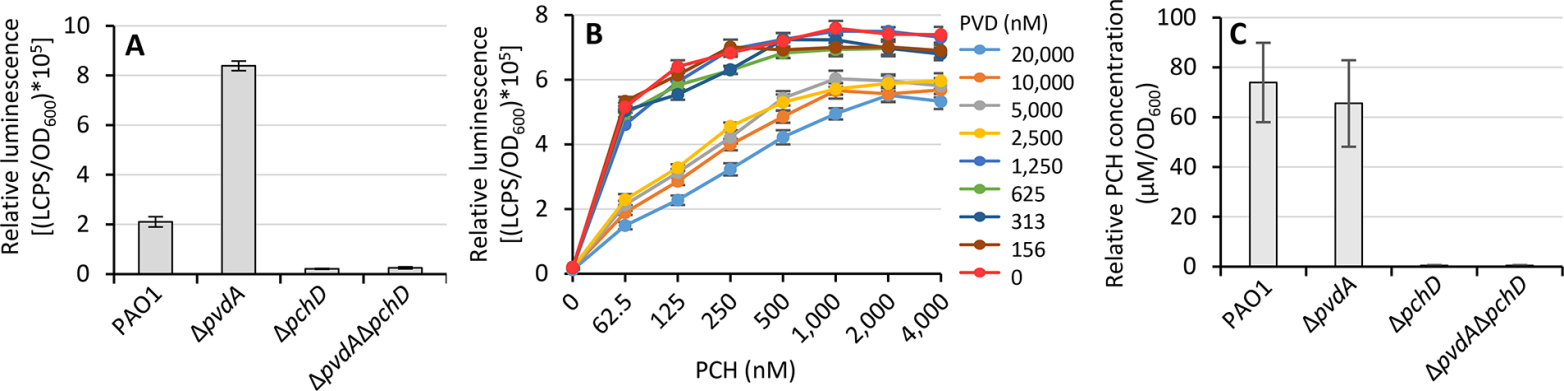
Factors affecting PCH detection in *P. aeruginosa* culture supernatants. Relative bioluminescence emission of the *P. aeruginosa* Δ*pvdA*Δ*pchD*Δ*fpvA* P*pchE::lux* biosensor, in the presence of PAO1, Δ*pvdA*, Δ*pchD*, and Δ*pvdA*Δ*pchD* culture supernatants obtained after a 24 h growth in DCAA (A) or in the presence of increasing concentrations of PCH (from 0.625 to 4 μM) and of PVD (from 0.625 to 20 μM) (B). (C) PCH concentrations (μM/OD_600_) in the diluted (1:1024) supernatants of the indicated *P. aeruginosa* strains grown for 24 h in DCAA. Data are representative of three independent experiments ± SD.

The ability of the biosensor to specifically and rapidly respond to low PCH concentrations encouraged its use for direct PCH quantification in *P. aeruginosa* culture supernatants. Calibration of the biosensor can easily be obtained with commercially available PCH (Santa Cruz Biotechnology Inc., CA). To this purpose, the supernatants of wild-type PAO1 and Δ*pvdA* and Δ*pchD* single and double mutants were collected after 24 h of growth in the iron-poor medium DCAA, and the biosensor bioluminescence emission was measured. As expected, no bioluminescence emission was recorded in culture supernatants of PCH-defective mutants, indicating that no compound other than PCH can trigger the biosensor (Figure 4A). Surprisingly, the bioluminescence emission of the PAO1 culture supernatant was much lower than that of the *pvdA* mutant (Figure 4A). This result is in contrast with previous data showing that wild-type PAO1 and PVD defective mutants produce a similar amount of PCH under iron-limited conditions^24^ and suggests that high PVD levels can interfere with the biosensor response. To verify this hypothesis, PCH was extracted from the supernatant of PAO1, Δ*pvdA*, Δ*pchD*, and Δ*pvdA*Δ*pchD* and analyzed by TLC. The PCH spot of PAO1 was quantitatively similar that of the *pvdA* mutant (Figure S4A), suggesting that PCH production in these two strains is comparable under the test conditions. Since PAO1 produces a high amount of PVD when grown in DCAA (Figure S4B), we hypothesized that PVD may hamper PCH measurements by chelating iron traces in the growth medium. Indeed, previous work has demonstrated that PchR binds PCH in its iron-loaded form, while it does not in its apo-form.^21,58^ Therefore, iron withholding by PVD could negatively affect PCH gene expression, and consequently bioluminescence emission. To verify this hypothesis, the biosensor response was measured in the presence of increasing concentrations of purified PCH and PVD (62.5–4000 and 20–20,000 nM, respectively). The choice of using 20,000 nM as the maximum PVD concentration derives from the experimental observation that *P. aeruginosa* PAO1 produces ≈ 200 μM PVD in DCAA culture supernatants (Figure S4B) and that a 1:10 dilution (*i.e.*, 5 μL of culture supernatants added to 45 μL of biosensor suspension) is used for the assay. PVD concentrations of ≥2500 nM significantly reduced the biosensor luminescence emission (Figure 4B), even in the presence of elevated PCH concentrations. Conversely, PVD concentrations of ≤1250 nM had a negligible effect on the biosensor luminescence emission at all PCH concentrations tested. The above findings suggest that an appropriate dilution of *P. aeruginosa* culture supernatants can overcome PVD interference in the PCH biosensor assay. To corroborate these results, the culture supernatants of wild-type PAO1 and Δ*pvdA* and Δ*pchD* single and double mutants were serially diluted, and the bioluminescence emission of the Δ*pvdA*Δ*pchD*Δ*fpvA* P*pchE::lux* biosensor was measured after 3.5 h at 25 °C. Interestingly, the luminescence emission progressively increased with serial dilution of the PAO1 supernatant, reaching the same values of the PVD-deficient Δ*pvdA* mutant at 1:32 dilution (Figure S5A). Moreover, the biosensor luminescence emission in the presence of the Δ*pvdA* mutant supernatant was nearly constant up to the 1:32 dilution, suggesting that PCH levels in supernatants of the PVD-defective mutant exceeded the upper detection limit of the biosensor, resulting in its saturation.

The above results indicate that an appropriate dilution of culture supernatants is mandatory for PCH quantification. To calculate the concentration of PCH produced by *P. aeruginosa*, 1:256, 1:512, and 1:1024 dilutions were used, and the PCH concentration was calculated by using a standard calibration curve (Figure S5B). Interestingly, the PCH concentrations estimated by using the three different dilutions gave comparable results (Figure S5C), as expected for PCH concentrations below the biosensor saturation point (*i.e.*, 160 nM, Figure 2I and Figure 3C). Therefore, the lowest sample dilution (1:1024) was selected for further experiments (see also the Supporting Information, text S1). By diluting the supernatant, the PCH concentrations determined for PAO1 and the Δ*pvdA* mutant culture supernatants (≈70 μM/OD_600_) were in line with previous experiments (Figure 4C) and literature data.^59^

### PCH Quantification in *P. aeruginosa* Clinical Strains and Biological Fluids

**Figure 5:**
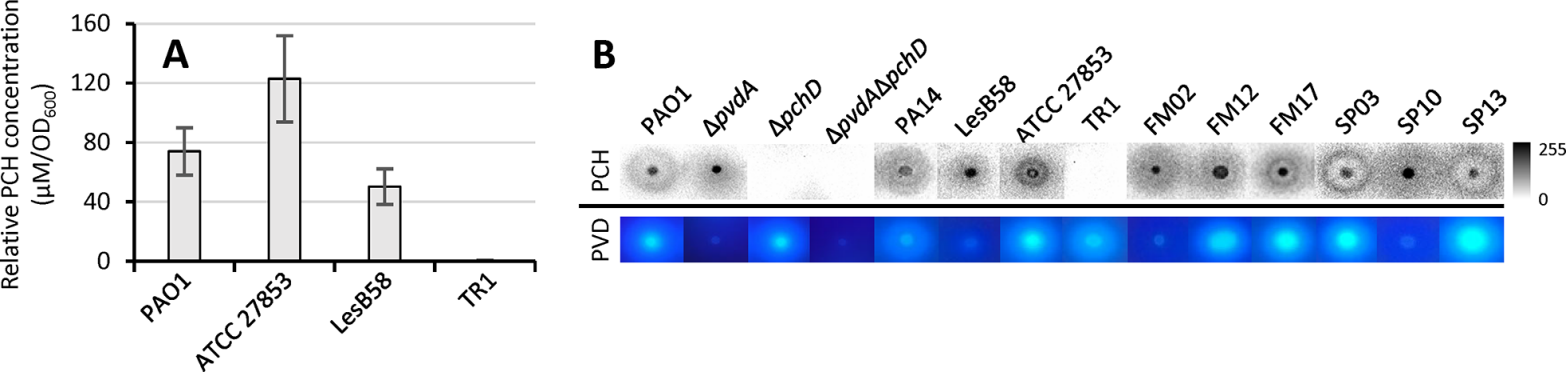
Bioassays for PCH detection in liquid and solid media. (A) PCH concentrations (μM/OD_600_) in the culture supernatants of *P. aeruginosa* strains PAO1, ATCC 27853, LesB58, and TR1 grown in DCAA for 24 h. Supernatants were 1:1024 diluted, and PCH concentration was calculated using a PCH calibration curve, as outlined in the Supporting Information, text S1. Data are representative of three independent experiments ± SD. (B) Top: qualitative detection of PCH production on CAA agarose plates, as outlined in the Supporting Information, text S2. *P. aeruginosa* strains were spotted on CAA agarose plates, grown for 24 h at 37 °C, and killed by chloroform vapor. Then, CAA agarose plates were overlaid with the *P. aeruginosa* Δ*pvdA*Δ*pchD*Δ*fpvA* P*pchE::lux* biosensor strain and incubated for 1 h at 37 °C. The PCH grey halo was detected using a ChemiDoc XRS+ Imaging System (Bio-Rad). The gray scale denotes pixel intensity. Bottom: PVD production by the *P. aeruginosa* strain, prior to the biosensor overlay, detected as fluorescence emission upon UV light exposure. Images are representative of one of several independent tests providing similar results.

To check the ability of the biosensor to measure PCH levels produced by clinical *P. aeruginosa* strains, *P. aeruginosa* ATCC 27853^60^ and the Liverpool-epidemic strain LesB58^61^ were preliminarily tested. *P. aeruginosa* ATCC 27853 and LesB58 produce different PVD types (*i.e.*, type II and III PVD, respectively) and release quite different PVD levels in the medium (Figure S4B). *P. aeruginosa* strains were cultured for 24 h in DCAA, and the supernatants were diluted. The biosensor response to the supernatant of ATCC 27853 was similar to that observed with PAO1, showing biosensor saturation and PVD interference for ≤1:32 supernatant dilution. Contrarily, the supernatant of LesB58 determined an initial increase (2-fold dilution) of bioluminescence emission, which remained constant up to the 64-fold dilution (Figure S5D). The different response profile can be ascribed to the different PVD concentrations in the supernatants of two test strains (Figure S4B). Indeed, the amount of PVD produced by PAO1 and ATCC 27853 under iron-poor conditions is almost the same (∼200 μM), while the PVD concentration in the LesB58 supernatant is 10-fold lower (Figure S4B). These findings further corroborate the need of eliminating PVD interference by diluting the supernatant prior to PCH measurement. On this basis, to calculate the exact concentration of PCH produced by PAO1, ATCC 27853, and LesB58, culture supernatants were diluted 1:1024, and 5 μL of each supernatant were used for the assay, as recommended in the Supporting Information, text S1. Preliminary dilution lowers the PVD concentration to <40 nM, thereby preventing any interference of PVD with biosensor response to PCH (Figure 4B). The clinical *P. aeruginosa* isolate TR1 was included as a negative control, being a natural PCH-defective mutant.^62^ Interestingly, PCH production was slightly lower in *P. aeruginosa* LesB58 than in ATCC 27853 and PAO1, while no PCH was detected in the supernatant of the PCH-negative strain TR1 (Figure 5A). Altogether, these results indicate that, with an appropriate setting (Supporting Information, text S1), the biosensor is a valuable tool to quantify PCH production by *P. aeruginosa* clinical isolates.

Lastly, the newly generated whole-cell biosensor was adapted to generate a rapid screening system of PCH-producing *P. aeruginosa* strains on solid media. To this aim, several *P. aeruginosa* strains (listed in Table S1) were spot-inoculated on CAA agarose plates and grown at 37 °C for 24 h until ∼3 mm colonies became visible. Colonies were inspected under UV light exposure to detect PVD production (blue halo), and then bacteria were killed with chloroform vapors. Thereafter, a thin (∼1 mm) agarose layer containing the Δ*pvdA*Δ*pchD*Δ*fpvA* P*pchE::lux* biosensor was overlaid onto the test colonies and bioluminescence emission was detected after 1 h incubation at 37 °C. To validate the plate-based screening system, wild-type *P. aeruginosa* PAO1 and PCH and/or PVD defective mutants were tested. Noteworthy, the same signal was registered for wild-type *P. aeruginosa* PAO1 and PVD-null mutant Δ*pvdA*, (Figure 5B), while no light emission was observed for Δ*pchD*, Δ*pvdA*Δ*pchD*, and TR1 PCH-null mutants (Figure 5B). Although with some differences, probably due to growth rate variations and PVD production levels (Figure 5B, bottom), all clinical strains tested positive for PCH production (Figure 5B).

The PCH plate assay was then applied to the screening of 98 *P. aeruginosa* clinical isolates from CF patients at different stages of lung infection. The vast majority of isolates produced both siderophores (Table S4); PVD- and PCH-negative isolates were only 16.3 and 7.1%, respectively, with two isolates testing double-negative.

**Figure 6:**
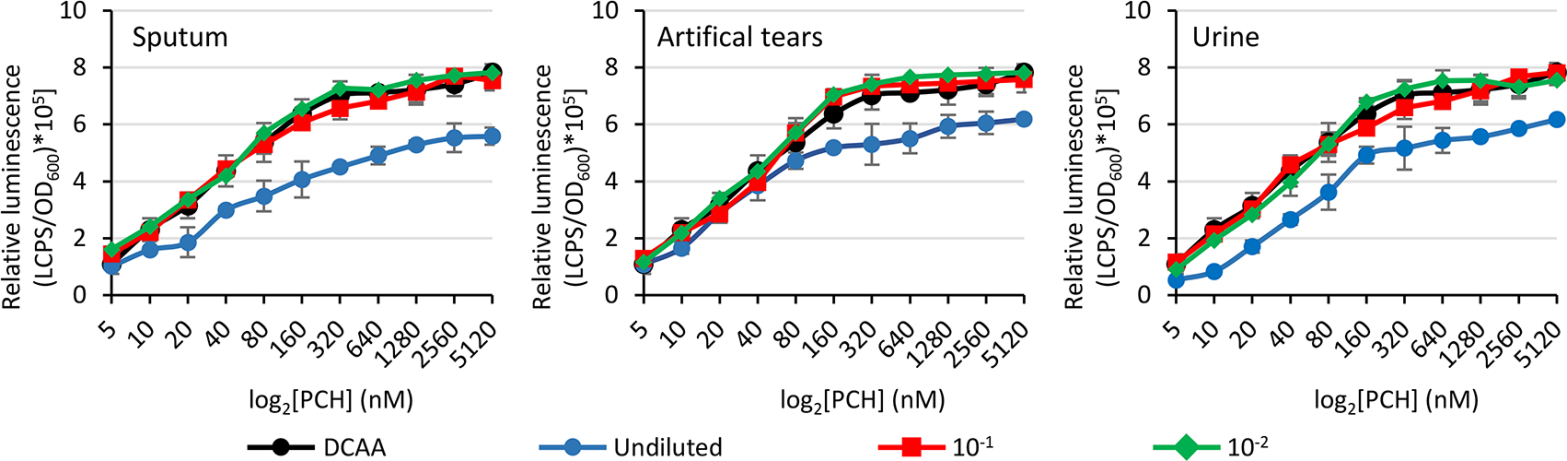
Biosensor response to PCH-spiked biological fluids. Relative light emission (LCPS/OD_600_) of the *P. aeruginosa* Δ*pvdA*Δ*pchD*Δ*fpvA* P*pchE::lux* biosensor in response to increasing concentrations of PCH (5 to 5120 nM) in undiluted and diluted (10^–1^ and 10^–2^) biological fluids, after 3.5 h of incubation at 25 °C. Each value represents the mean ± SD of three independent experiments.

The biosensor performances were also tested using sputum, artificial tears, and urine as input samples, upon spiking with known PCH concentrations (5 to 5120 nM). While undiluted fluids significantly reduced the biosensor response, negligible interference was observed for 10- and 100-fold diluted fluids (Figure 6).

Interestingly, transcription of *pch* biosynthetic genes was previously demonstrated in the majority of CF sputa, though PCH was detected in only 13% of them, likely due to the poor sensitivity of the PCH detection method (∼1 μM).^26^ Here, pilot testing of sputa from eight anonymous CF patients revealed that PCH was detectable in six of them (range 86.9 ± 4.6–24,390.0 ± 91.2 nM), showing an excellent correlation between the presence of PCH and *P. aeruginosa* culture-positivity of sputa (Table S5).

Altogether, these results indicate that the Δ*pvdA*Δ*pchD*Δ*fpvA* P*pchE::lux* biosensor is suitable for PCH quantification in both *P. aeruginosa* culture supernatants and biological fluids and can be used for the rapid screening of PCH-producing *P. aeruginosa* isolates on a solid medium (Supporting Information, text S2).

## Conclusions

In summary, we generated a whole cell-based biosensor for PCH quantification and tested its proficiency under laboratory conditions. The biosensor specificity is guaranteed by the unique selectivity of the PCH transport machinery and the PchR activator of the P*pchE::lux* reporter fusion. In fact, both the FptA receptor and the PchR regulator are strictly PCH-selective.^58,63^ Accordingly, evidence was provided that the biosensor does not respond to PCH stereoisomers or precursors, as well as to a variety of iron-chelating compounds, excluding the possibility of false positive readouts.

Two main factors, namely, iron and PVD carryover in the test sample, were shown to interfere with the biosensor performances. Both factors, however, do not undermine the biosensor validity by virtue of the extremely high sensitivity of the assay (lower detection limit = 1.64 ± 0.26 nM PCH), which requires the sample to be diluted several fold (≅10^–3^) prior to testing, so that interference by iron and PVD would be negligible. It should also be taken into account that PCH is produced by *P. aeruginosa* only under conditions of severe iron limitation [<5 μM Fe(III)],^40^ implying that the presence of sufficient (≥ 5 μM) iron levels in biological samples would be incompatible with PCH production by *P. aeruginosa*. In practice, considering that the concentration of secreted PCH is extremely variable depending on the *P. aeruginosa* strain and growth conditions (from ∼800,000 to ∼1000 nM in optimized laboratory media and CF sputa, respectively),^11,34^ appropriate serial dilutions of the sample should be made to adjust the PCH concentration in the linear response range of the biosensor (5–40 nM; Figure 3A). Of note, a calibration curve can be easily prepared using pure PCH, which is commercially available.

The luminescent biosensor offers several advantages over previous PCH detection and/or quantification methods, which rely on chromatographic or spectrophotometric analysis of partially purified organic extracts. Briefly, (i) the assay protocol is straightforward and allows PCH to be quantified in 3.5 h with negligible sample handling; (ii) up to 28 samples can contemporarily be tested in triplicate using a single 96-well microplate, also including standard samples for system calibration; (iii) the microtiter plate format would allow the assay to be scaled down to a smaller format for higher throughput (*e.g.*, using 384-well microplates) and is amenable to automation (*e.g.*, using automatic dispensers); (iv) once dispensed with the reporter strain, the microtiter plates can be stored frozen at −80 °C for months with negligible loss of the assay performances (Figure S6); (v) the assay combines high sensitivity (LOQ in the nM PCH range) with simplicity, since the luciferase-based reporter system is characterized by a high signal/noise ratio and does not require an exogenous substrate for signal emission. Bioluminescence background levels in living cells are extremely low, making bioluminescence up to 50 times more sensitive than fluorescence, which would be impracticable in testing the intrinsically fluorescent *Pseudomonas* species.^52,64^ Moreover, *P. luminescens* luciferase is endowed with remarkable chemical and physical stability.^65^

The inclusion of *P. aeruginosa* into the list of risk group 2 bacterial pathogens could represent a limitation to the use of the luminescent biosensor in biosafety level 1 laboratories. However, the *P. aeruginosa* PAO1 Δ*pvdA*Δ*pchD*Δ*fpvA* mutant carries stable genetic knock-outs of both PCH and PVD biosynthesis genes, together with a deletion of the PVD receptor gene. Altogether, these mutations impair iron uptake and result in an avirulent phenotype in animal models of infections,^24,66,67^ downgrading the risk associated with biosensor manipulation.

In conclusion, the Δ*pvdA*Δ*pchD*Δ*fpvA* P*pchE::lux* whole cell-based biosensor represents an innovative tool to detect and quantify the PCH siderophore. The preliminary setup of the test conditions allows the fast, easy, accurate, and cost-effective determination of nanomolar PCH concentrations in *P. aeruginosa* liquid cultures and biological fluids, as well as the qualitative screening of PCH-producing *P. aeruginosa* colonies on an agar plate, hopefully facilitating future investigations on the role of PCH in the pathogenesis of *P. aeruginosa* infection.

## Supporting Information Available

Supplementary methods, figures, tables, and description of the analytical protocol (PDF)

## ABBREVIATIONS

CAA: casamino acids
DCAA: deferrated casamino acids
DFP: deferiprone
DFO: deferoxamine
DMSO: dimethyl sulfoxide
Fur: ferric uptake regulator protein
HPLC: high-performance liquid chromatography
LB: Luria-Bertani broth
LCPS: light counts per second
LOD: limit of detection
LOQ: limit of quantification
PVD: pyoverdine
PCH: pyochelin
S: slope of calibration curve
SAL: salicylate
S/N: signal/noise ratio
SD: standard deviation
TLC: thin layer chromatography
