# Supplementary material for: A Highly Sensitive Luminescent Biosensor for the Microvolumetric Detection of the Pseudomonas aeruginosa Siderophore Pyochelin
Source: ACS Sens. 2021 Sep 3;6(9):3273–83. doi: 10.1021/acssensors.1c01023 (PMC8477383; doi:10.1021/acssensors.1c01023)
Supplement: Supplementary file 1 — se1c01023_si_001.pdf [file se1c01023_si_001.pdf]

SUPPLEMENTARY INFORMATION

A highly sensitive luminescent biosensor for the microvolumetric detection of the *Pseudomonas aeruginosa* siderophore pyochelin

Daniela Visaggio[ab*], Mattia Pirolo[a], Emanuela Frangipani[c], Massimiliano Lucidi[a], Raffaella Sorrentino[d], Emma Mitidieri[d], Francesca Ungaro[e], Andrea Luraghi[f], Francesco Peri[f] and Paolo Visca[ab*]

[a]Department of Science, Roma Tre University, 00146, Rome, Italy

[b]Santa Lucia Fundation IRCCS, 00179, Rome, Italy

[c]Department of Biomolecular Sciences, University of Urbino Carlo Bo, 61029, Urbino, Italy

[d]Department of Molecular Medicine and Medical Biotechnology, School of Medicine, University of Naples Federico II, 80138, Naples, Italy

[e]Department of Pharmacy, University of Naples Federico II, 80131, Naples, Italy

[f]Department of Biotechnology and Biosciences, University of Milano-Bicocca, 20126, Milan, Italy

*Corresponding Authors: Paolo Visca, paolo.visca@uniroma3.it; Department of Science, Roma Tre University, Viale Guglielmo Marconi 446, 00146 Rome, Italy; Phone: +39.06.57336347 - Daniela Visaggio daniela.visaggio@uniroma3.it; Department of Science, Roma Tre University, Viale Guglielmo Marconi 446, 00146 Rome, Italy; Phone: +39.06.57336408.

Table of contents

Figure S1	Synthesis of (L) and (D)-N-Methyl cysteine	ii
Figure S2	Synthesis of both PCH (5) and enantio-PCH (6) from the same precursors	iii
Figure S3	Growth and PVD production by different *P. aeruginosa* strains in DCAA supplemented with increasing iron concentrations	iv
Figure S4	PCH and PVD production by different *P. aeruginosa* strains	v
Figure S5	PCH quantification in *P. aeruginosa* culture supernatants	vi
Figure S6	PCH quantification in *P. aeruginosa* culture supernatants before and after biosensor storage at -80°C	vii
Table S1	Strains and plasmids used in this study	viii
Table S2	Oligonucleotides used in this study	ix
Table S3	Compounds used in this study	x
Table S4	Antibiotic-resistance profile and siderophore production by *P. aeruginosa* isolates from sputa of CF patients at different stages of lung infection	xi
Table S5	PCH detection in sputum samples from CF patients	xiii
Supplemental text S1	Protocol for quantification of PCH in *Pseudomonas aeruginosa* culture supernatants and CF sputa by means of the *P. aeruginosa* Δ*pvdA*Δ*pchD*Δ*fpvA* P*pchE::lux* biosensor	xiv
Supplemental text S2	Protocol for the detection of PCH production on solid agar plates by means of the *P. aeruginosa* Δ*pvdA*Δ*pchD*Δ*fpvA* P*pchE::lux* biosensor	xviii
Graphical workflow	Schematic illustration of the steps required for PCH detection on solid medium and PCH quantification from liquid samples	xxi
References		xxii

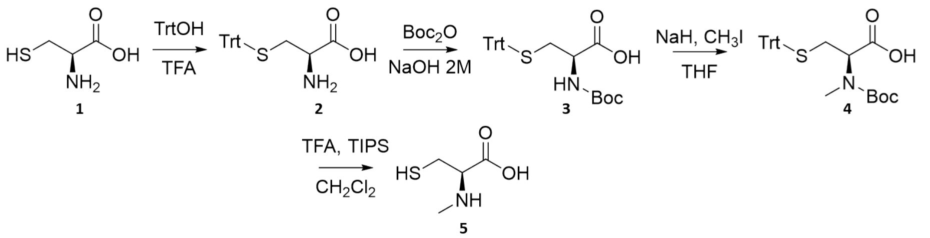

Figure S1. Synthesis of (L) and (D)-N-Methyl cysteine. In order to produce S-trityl-L-cysteine (**2**), Triphenylmetanol (1 eq.) was added to a solution of L-Cys (**1**, 1 eq.) in 10 mL of TFA. After stirring overnight at room temperature Et_2O was added and the pH was adjusted to 5-6 by addition of aq. 4M NaOH and 10% sodium acetate. The resulted solid precipitate S-trityl-L-cysteine (**2**) was collected by filtration and washed with Et_2O. The product was used as so, without further purification [yield 98%.^{1}H NMR (400 MHz, DMSO-d_6) δ 7.43 – 7.12 (m, 14H), 2.89 (dd, J = 9.2, 4.3 Hz, 1H), 2.55 (dd, J = 12.5, 4.3 Hz, 1H), 2.36 (dd, J = 12.5, 9.3 Hz, 1H)].

S-trityl-D-cysteine was synthesized with the same procedure starting from D-Cys [Yield 94%. ^{1}H NMR (400 MHz, DMSO-d_6) δ 7.31 (d, J = 5.9 Hz, 11H), 2.91 (d, J = 8.7 Hz, 1H), 2.56 (d, J = 13.8 Hz, 1H), 2.39 (t, J = 10.9 Hz, 1H)].

To produce N-(tert-butoxycarbonyl)-S-trityl-L-cysteine (**3**), (1 eq.), 7 mL of aq. 2M NaOH was added to the precipitate **2** and then Boc_2O (1.7 eq.) was added dropwise at 0°C. After stirring 24h at room temperature the solution was treated with aq. 1M HCl until pH 2, the resulting mixture was extracted with CH_2Cl_2. The combined organic phases were washed with brine, dried over Na_2SO_4 and evaporated to dryness, to afford the desired product as a white solid [yield 93%. ^{1}H NMR (400 MHz, Chloroform-d) δ 7.48 – 7.11 (m, 15H), 5.00 (d, J = 8.0 Hz, 1H), 4.22 (d, J = 7.1 Hz, 1H), 2.65 (d, J = 5.4 Hz, 2H), 1.44 (s, 9H)].

N-(tert-butoxycarbonyl)-S-trityl-D-cysteine was synthesized with the same procedure starting from S-trityl-D-cysteine [yield 94 %. ^{1}H NMR (400 MHz, Chloroform-d) δ 7.46 – 7.21 (m, 15H), 4.88 (s, 1H), 4.05 (s, 1H), 2.69 (d, J = 5.7 Hz, 2H), 1.45 (d, J = 8.3 Hz, 9H)]. In order to synthesis N-(tert-butoxycarbonyl)-N-methyl-S-trityl-L-cysteine (**4**), **3** was added carefully at 0°C to a suspension of NaH (2.4 eq.) in THF under Ar atmosphere. After 30 min MeI (8 eq) was added dropwise at 0°C. The resulting solution was stirred at room temperature for 24h. After addition of water at 0°C the solution was allowed to stir for 30 min. The product was concentrated in vacuo and the pH was adjusted to 5 by the addition of 6M HCl. The aqueous phase was extracted with CH_2Cl_2 dried over Na_2SO_4 and evaporated to dryness. Crude extract was purified by flash chromatography (EtOAc: petroleum spirit 4:6 + 0.3% AcOH) to afford the desired product as a clear oil [yield 33%. ^{1}H NMR (400 MHz, Chloroform-d) δ 7.47 – 7.04 (m, 15H), 3.54 (s, 1H), 2.91 (d, J = 10.6 Hz, 1H), 2.45 (s, 3H), 2.27 (t, J = 11.0 Hz, 1H), 1.27 (d, J = 6.9 Hz, 9H)]. N-(tert-butoxycarbonyl)-N-methyl-S-trityl-D-cysteine was synthesized with the same procedure starting from N-(tert-butoxycarbonyl)-S-trityl-D-cysteine [yield 30%. ^{1}H NMR (400 MHz, Chloroform-d) δ 7.46 – 7.15 (m, 15H), 3.83 (d, J = 7.8 Hz, 1H), 3.69 (s, 1H), 2.78 (d, J = 10.3 Hz, 1H), 2.68 (d, J = 9.0 Hz, 3H), 1.41 (d, J = 26.9 Hz, 9H)]. To a solution of **4** (1 eq.) in CH_2Cl_2, TFA (26 eq.) and TIPS (5 eq.) were added causing the solution colour to turn yellow. After stirring for 4 h at room temperature, the solvents were evaporated in vacuo, water was added to the crude extract and washed with petroleum spirit. Water phase was evaporated to dryness to afford the desired product as a white solid of N-methyl-L-cysteine (**5**) [yield 90%. ^{1}H NMR (400 MHz, D_2O) δ 3.97 (t, J = 4.3 Hz, 1H), 3.06 (dd, J = 15.4, 4.3 Hz, 1H), 2.92 (dd, J = 15.3, 4.5 Hz, 1H), 2.60 (d, J = 8.1 Hz, 4H)]. N-methyl-D-cysteine was synthesized with the same procedure starting from N-(tert-butoxycarbonyl)-N-methyl-S-trityl-D-cysteine [yield 84%. ^{1}H NMR (400 MHz, D_2O) δ 3.78 (t, J = 5.5 Hz, 1H), 3.12 (dd, J = 13.8, 4.8 Hz, 1H), 3.05 (dd, J = 13.8, 6.1 Hz, 1H), 2.71 (s, 3H)].

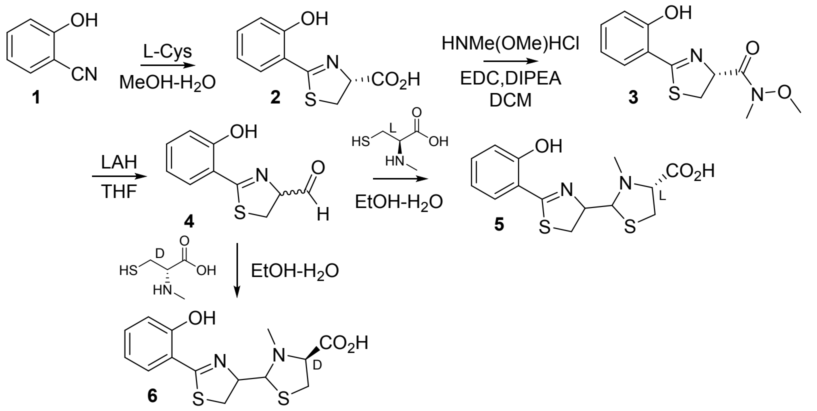

Figure S2. Synthesis of both PCH (5) and enantio-PCH (6) from the same precursors. To a solution of 2-hydroxybenzonitrile (**1**, 1 eq) in 3 mL of methanol was added L-Cys (2 eq) in 7 mL aq. Na_2HPO_4/NaH_2PO_4 pH 6.4 buffer solution adjusting the pH by aq. 6M HCl addition. The resulting mixture was refluxed for 48 h and then concentrated in vacuum until solid precipitation. The solid was suspended in water and the pH adjusted to 2 by solid citric acid addition. Aqueous phase was extracted with CH_2Cl_2, the combined organic layers were dried over Na_2SO_4 and evaporated to dryness. The product 2-(2-Hydroxyphenyl)-4,5-dihydro-thiazole-4-carboxylic acid (**2**) was obtained as yellow powder without any further purification [yield 91%. 1H NMR (400 MHz, DMSO-d6) δ 7.42 (t, J = 7.3 Hz, 2H), 7.06 – 6.82 (m, 2H), 5.42 (t, J = 8.5 Hz, 1H), 3.76 – 3.60 (m, 2H)]. Then a solution of the acid 2 (1 eq.) in 10 mL of CH_2Cl_2 and TEA (1.1 eq.), HNMe(OMe)HCl (1.1 eq.) and ECD (1.5 eq.) were added at 0°C. The reaction was allowed to stir overnight at room temperature then concentrated in vacuo. The resulting solid was suspended in EtOAc and washed with aq. 1M HCl solution. The organic phase was dried over Na_2SO_4 and evaporated to dryness. The crude 2'-(2-Hydroxyphenyl)-2'-thiazole-4'-(N-methoxy-N-methyl,) carboxamide (**3**) was purified by flash chromatography (EtOAc:Petroleum Spirit 3:7),[yield 76%. 1H NMR (400 MHz, Chloroform-d) δ 7.43 (dd, J = 7.8, 1.6 Hz, 1H), 7.36 (ddd, J = 8.6, 7.3, 1.6 Hz, 1H), 6.99 (dd, J = 8.3, 1.1 Hz, 1H), 6.93 – 6.80 (m, 1H), 5.70 (t, J = 9.1 Hz, 1H), 3.84 (s, 3H), 3.77 (d, J = 10.0 Hz, 1H), 3.49 (dd, J = 10.9, 9.2 Hz, 1H), 3.29 (s, 3H)]. The Weinreb amide (1 eq) was dissolved in 7 mL of THF under Ar atmosphere. LAH 1M in THF (1.3 eq.) was added dropwise at -40°C. After addition, the temperature was allowed to rise at -20 °C over 30 min and the solution stirred for additional 30 min. Twenty-five mL of Aq. sat NH_4Cl and 10 mL aq. 1M $KHSO_4$ was added dropwise at -20°C. After warming at room temperature, the solution was extracted with Et_2O. The combined organic layers dried over Na_2SO_4 were evaporated to dryness. The resulting compound (**4**) was used immediately for the following step without purification and characterization. The freshly synthetized aldehyde (1 eq) was dissolved under Ar in 13 mL of a 75/25 mixture EtOH/H_2O, then AcOK (6.5 eq.) and N-Me-L-Cys (Figure S1, 5) (3.5 eq.) was added at room temperature. The solution was stirred overnight at room temperature protected by the light. After dilution with water the pH was adjusted to 5 by the addition of solid citric acid. The aqueous phase was then extracted with EtOAc, the combined organic phases were dried over Na_2SO_4 and evaporated to dryness. The product (**5**) and (**6**) was obtained as mixture of four inseparable diastereoisomers without further purification [yield 25%. 1H NMR (400 MHz, Acetone-d6) δ 7.52 – 7.33 (m, 18H), 6.94 (t, J = 7.8 Hz, 20H), 5.30 (td, J = 9.1, 4.6 Hz, 1H), 5.22 (td, J = 8.9, 5.3 Hz, 2H), 5.04 (dd, J = 21.9, 6.5 Hz, 2H), 4.84 (q, J = 8.3 Hz, 4H), 4.59 (dd, J = 30.0, 6.8 Hz, 4H), 4.31 (dd, J = 32.5, 7.3 Hz, 6H), 4.04 (t, J = 6.0 Hz, 4H), 3.83 – 3.56 (m, 9H), 3.36 – 3.28 (m, 5H), 3.26 – 3.19 (m, 8H), 2.72 (s, 4H)].

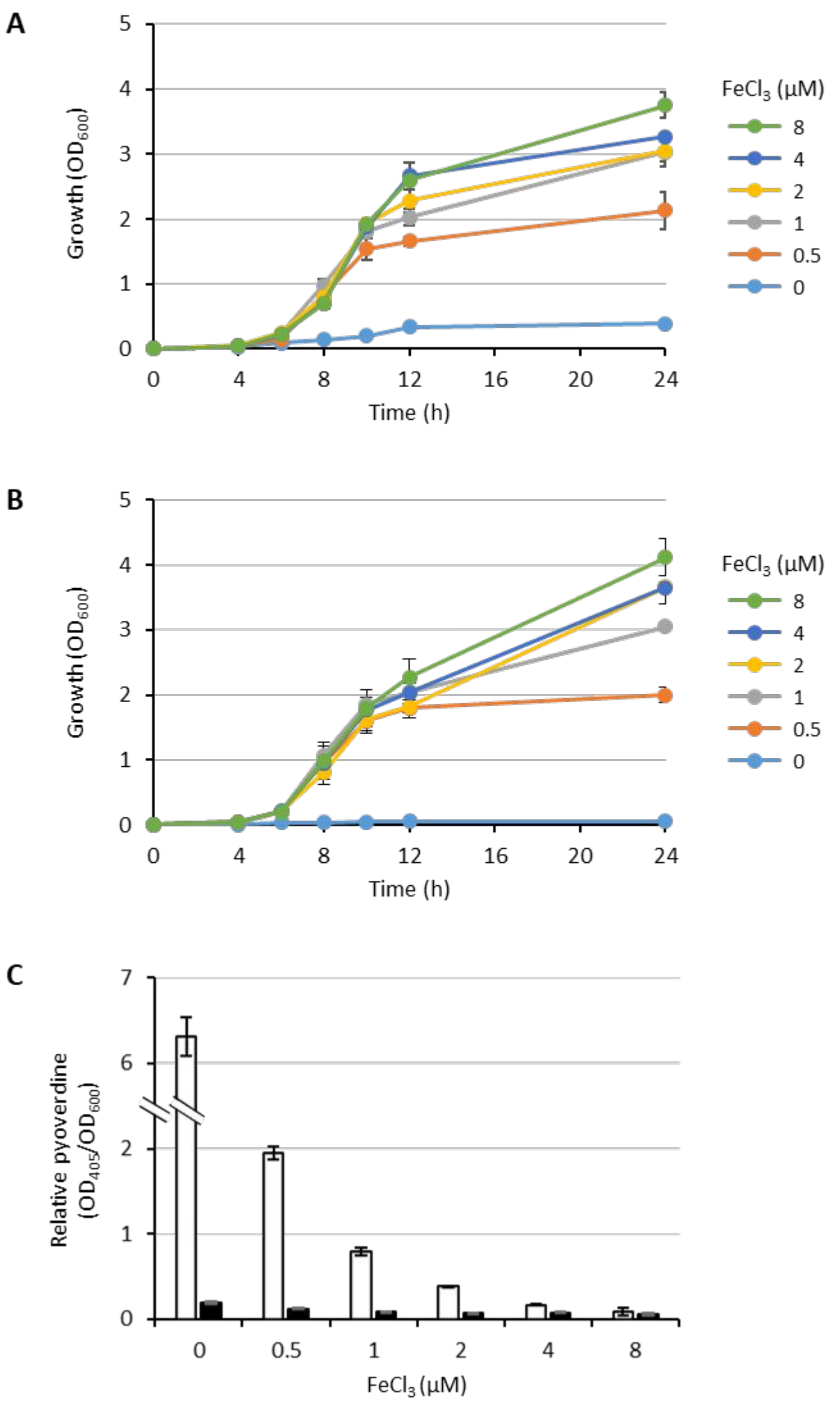

Figure S3. Growth and PVD production by different *P. aeruginosa* strains in DCAA supplemented with increasing iron concentrations. Growth of wild-type PAO1 (A) and of the Δ*pvdA*Δ*pchD*Δ*fpvA* triple mutant (B) in DCAA in the absence of iron or in the presence of increasing $FeCl_3$ concentrations, ranging from 0.5 to 8 µM. C) Pyoverdine production by PAO1 (white bars) and by the Δ*pvdA*Δ*pchD*Δ*fpvA* mutant (black bars) after 24-h growth in DCAA in the presence of increasing iron concentrations. Data are the mean of three independent experiments ± SD.

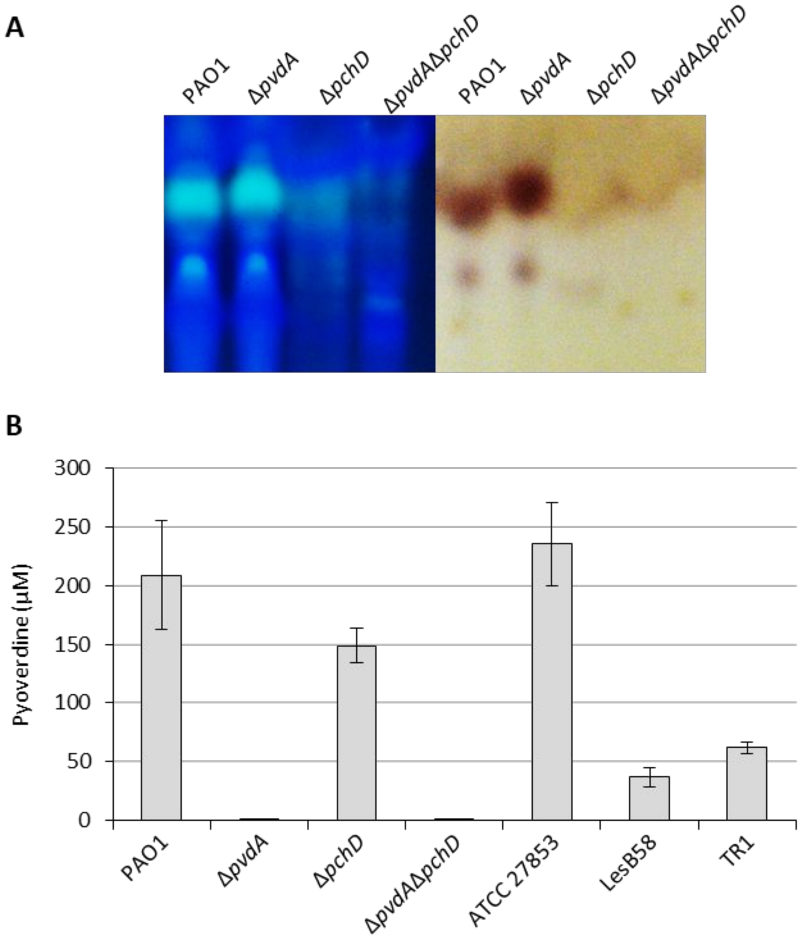

Figure S4. PCH and PVD production by different *P. aeruginosa* strains. A) PCH extracted from culture supernatants of *P. aeruginosa* PAO1, Δ*pvdA*, Δ*pchD*, and Δ*pvdA*Δ*pchD* after 24 h of growth in DCAA, and separated (5 µL of extract) by thin-layer chromatography (TLC). Chromatograms were visualized by exposure to UV light (left) and by spraying with 100 µM $FeCl_3$. B) PVD production by PAO1, Δ*pvdA*, Δ*pchD* and Δ*pvdA*Δ*pchD*, ATCC 27853, LesB58 and TR1. PVD was measured as the OD_{405} of *P. aeruginosa* supernatant after 24-h growth in DCAA at 37°C. Data are the mean of three independent experiments ± SD.

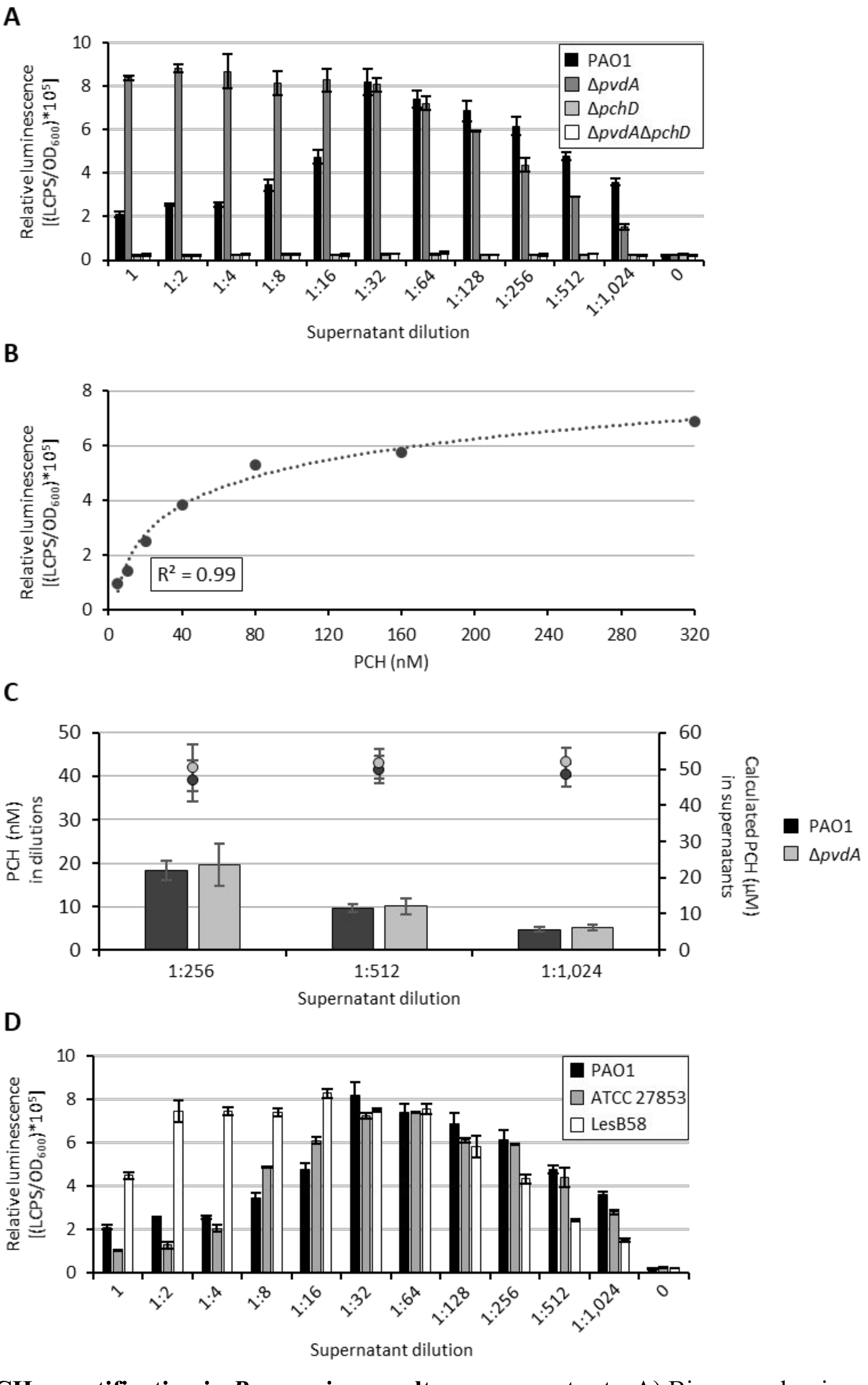

Figure S5. PCH quantification in *P. aeruginosa* culture supernatants. A) Biosensor luminescent emission expressed as LCPS/OD_{600} in response to serial dilutions of PAO1, Δ*pvdA*, Δ*pchD* and Δ*pvdA*Δ*pchD* culture supernatants. B) Example of a calibration curve determined by plotting the biosensor luminescent emission (LCPS/OD_{600}) *vs*. the PCH concentrations (5-320 nM). C) PCH concentration in culture supernatants of *P. aeruginosa* PAO1 and the Δ*pvdA* mutant grown for 24 h in DCAA. The left ordinate refers to histograms reporting the PCH concentration (nM) in diluted (1:256, 1:512 and 1:1,024) culture supernatants. The right ordinate refers to dots reporting the actual PCH concentration (µM) in the undiluted culture supernatants. D) Biosensor luminescent emission, expressed as LCPS/OD_{600}, in response to serial dilutions of PAO1, ATCC 27853 and LesB58 culture supernatants. The biosensor response was measured after 3.5-h incubation at 25 °C. Data are representative of three independent experiments ± SD.

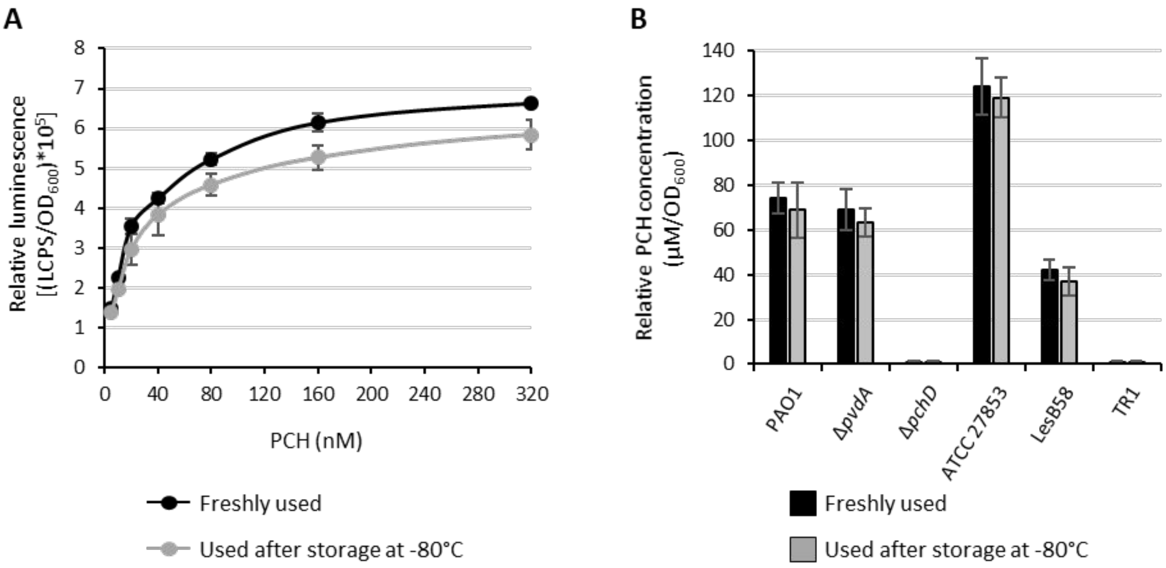

Figure S6. PCH quantification in *P. aeruginosa* culture supernatants before and after storage of the biosensor at -80° C. Biosensor was prepared according to Supplementary text S1. A) Dose-response plot of the biosensor immediately used (black line) or employed after one-month storage at -80°C (grey line). Relative light emission values (LCPS/OD_{600}) were taken after 3.5-h incubation at 25°C in presence of increasing PCH concentration (0-320 nM). B) PCH concentrations (μM/OD_{600}) in the culture supernatants of *P. aeruginosa* strains PAO1, Δ*pvdA*, Δ*pchD*, ATCC 27853, LesB58 and TR1, grown in DCAA for 24 h. Supernatants were 1:1,000 diluted and PCH concentration was calculated using the PCH calibration curve (panel A) as outlined in Supplementary text S1. The biosensor response in the presence of *P. aeruginosa* supernatants was measured after 3.5-h incubation at 25 °C. Data are representative of three independent experiments ± SD.

Table S1. Strains and plasmids used in this study

Strains	Relevant characteristics	Reference or source
E. coli		
DH5α	*recA1 endA1 hsdR17 supE44 thi-1 gyrA96 relA1* Δ*(lacZYA-argF)*U169 *[*φ80Δ*lacZ*ΔM15*]* F^- Nal^r	1
S17.1λ*pir*	Tp^R Sm^R *recA, thi, pro, hsdR*-M+RP4: 2-Tc:Mu:K_mTn7λ*pir*	2
P. aeruginosa		
PAO1 (ATCC 15692)		3
PAO1 Δ*pvdA*	PAO1 carrying a deletion in *pvdA*	4
PAO1 Δ*pchD*	PAO1 carrying a deletion in *pchD*	5
PAO1 Δ*pvdA*Δ*pchD*	PAO1 carrying a deletion in *pvdA* and *pchD*	6
PAO1 Δ*pvdA*Δ*pchD*Δ*fpvA*	PAO1 carrying a deletion in *pvdA*, *pchD* and *fpvA*	This study
PAO1 Δ*pvdA*Δ*pchD*Δ*fpvA* P*pchE::lux*	PAO1 Δ*pvdA*Δ*pchD*Δ*fpvA* containing the P*pchE::lux* transcriptional fusion integrated into the chromosome at the *attB* neutral site	This study
PAO1 Δ*pvdA*Δ*pchD*Δ*fpvA*Δ*pchR*	PAO1 Δ*pvdA*Δ*pchD*Δ*fpvA* carrying a deletion in Δ*pchR*	This study
PAO1 Δ*pvdA*Δ*pchD*Δ*fpvA*Δ*pchR* P*pchE::lux*	PAO1 Δ*pvdA*Δ*pchD*Δ*fpvA*Δ*pchR* containing the P*pchE::lux* transcriptional fusion integrated into the chromosomal neutral site *attB*	This study
ATCC 27853	Clinical isolate	7
PA14	Clinical isolate	8
LesB58	Clinical isolate	9
FM02	Clinical isolate	10
TR1	Clinical isolate	11
FM12	Clinical isolate	10
FM17	Clinical isolate	10
SP03	Clinical isolate	12
SP10	Clinical isolate	12
SP13	Clinical isolate	12
**Plasmids**		
pDM4	Suicide vector; *sacBR*, *oriR6K*; Cm^R	13
pME3087	Suicide vector; Tc^R	14
pDM4Δ*fpvA*	pDM4 derivative for *fpvA* deletion	This study
pMEΔ*pchR*	pME3087 derivative for *pchR* deletion	This study
mini-CTX-lux	Integration-proficient promoter probe plasmid *P. aeruginosa* vector, Tc^R	15
mini-CTX P*pchE::lux*	mini-CTX-lux-derived promoter probe plasmid carrying the P*pchE::luxCDABE* transcriptional fusion.	This study
pFLP2	FRT recombination vector with SacB counterselection	16

Table S2. Oligonucleotides used in this study

Oligonucleotides	Sequence (5' → 3')[a]	Restriction site	Application
fpvA mut_UP_FW	5'-ACGCGTCGACGACGACCTGGTCCAGGG-3'	SalI	Generation of the pDM4Δ*fpvA* construct
fpvA mut_UP_RV	5'-CCGGAATTCTGAGACCGTGTGGTGCTG-3'	EcoRI	Generation of the pDM4Δ*fpvA* construct
fpvA mut_DOWN_FW	5'-CCGGAATTCGAACCTGATGTTCAGCACTC-3'	EcoRI	Generation of the pDM4Δ*fpvA* construct
fpvA mut_DOWN_RV	5'-GCTCTAGAGGGTGGAACTGCTCGGTG-3'	XbaI	Generation of the pDM4Δ*fpvA* construct
pchR mut_UP_FW	5'-CGCGGATCCTGGTACAGCGGTGCCA-3'	BamHI	Generation of the pME3087Δ*pchR* construct
pchR mut _UP_RV	5'-CCGGAATTCATCAGGTTTTCCTGTAGC-3'	EcoRI	Generation of the pME3087Δ*pchR* construct
pchR mut_ DOWN_ FW	5'-CCGGAATTCCTGAGTCTCCGCGACGAC-3'	EcoRI	Generation of the pME3087Δ*pchR* construct
pchR mut_DOWN_RV	5'-CCCAAGCTTGCCCAGCACCTCGCC-3'	HindIII	Generation of the pME3087Δ*pchR* construct
P*pchE*_FW	5'-GCCAAGCTTGCTCTGCGACGAAGAGG-3'	HindIII	Costruction of the P*pchE::lux* transcriptional fusion
P*pchE*_RV	5'-CCCGGATCCATGGGGGCTCCCT-3'	BamHI	Costruction of the P*pchE::lux* transcriptional fusion

[a]Restriction sites are underlined

Table S3. Compounds used in this study

Compound abbreviation	Compound Name	Chemical Structure
PCH	Pyochelin	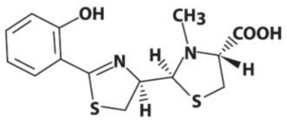
Enantio-PCH	Enantio-pyochelin	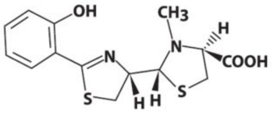
SAL	Salicylate	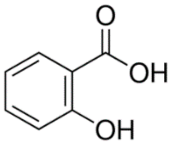
PVD	Pyoverdine	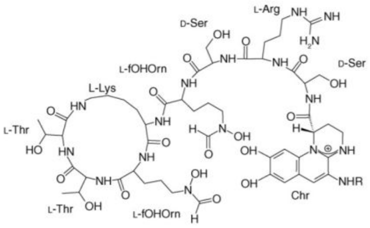
DFP	Deferiprone	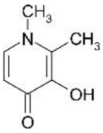
DFO	Deferoxamine	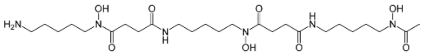

Table S4. Antibiotic-resistance profile and siderophore production by *P. aeruginosa* isolates from sputa of CF patients at different stages of lung infection

Isolate	Years of chronic CF lung colonization	R-Profile	PVD (OD_{405}/OD_{600})[a]	PCH production[b]
BG 1	First isolate	ATM, TM, LV	8.19	-
BG 2	3	ATM, TM, AK	0.78	+
BG 3	6	PIP/TAZ, ATM, CAZ, TM	1.58	-
BG 4	First isolate	ATM	3.44	+
BG 5	2	ATM, TM	2.32	+
BG 6	6	TM	0.75	+
BG 7	First isolate	ATM	2.05	+
BG 8	2	ATM, AK	2.19	+
BG 9	5	Susceptible	1.14	+
BG 10	First isolate	ATM	1.22	+
BG 11	3	ATM	1.39	+
BG 12	5	Susceptible	1.44	+
BG 13	First isolate	PIP/TAZ, ATM, CAZ, MEM, IMP, TM, CIP, LV	1.87	+
BG 14	3	ATM, CAZ, MEM, IMP, TM, LV	4.05	+
BG 15	5	ATM, CAZ, MEM, IMP, AK, TM	0.93	+
BG 16	First isolate	ATM	1.71	+
BG 17	2	ATM, AK	1.58	+
BG 18	6	AK	2.52	+
BG 19	First isolate	MEM, IMP, AK, TM	0.87	+
BG 20	3	MEM, IMP, AK, TM	1.09	-
BG 21	5	MEM, IMP, AK, TM	1.58	+
BG 22	First isolate	Susceptible	1.36	+
BG 23	2	Susceptible	2.09	+
BG 24	First isolate	CAZ, MEM, IMP, AK, TM, CIP, LV	0.01	-
BG 25	2	CAZ, MEM, IMP, AK, TM, CIP, LV	1.36	+
BG 26	5	PIP/TAZ, ATM, CAZ, MEM, IMP, AK, TM, CIP, LV	0.47	+
BG 27	First isolate	ATM	3.02	+
BG 28	2	ATM	1.11	+
BG 29	5	CIP, LV	2.15	+
BG 30	First isolate	ATM	0.26	+
BG 31	3	ATM	7.01	+
BG 32	5	ATM	3.64	+
BG 33	First isolate	ATM, AK	5.05	+
BG 34	2	ATM, AK, TM	6.72	+
BG 35	First isolate	ATM	0.44	+
BG 36	3	Susceptible	5.47	+
BG 37	First isolate	ATM, TM	3.24	+
BG 38	3	AK, TM, CIP	4.24	+
BG 39	First isolate	ATM	0.02	+
BG 40	3	MEM, IMP	0.96	-
BG 41	First isolate	ATM	0.01	+
BG 42	3	ATM, MEM, IMP	1.43	+
BG 43	First isolate	ATM	0.63	+
BG 44	3	CAZ, AK, TM	0.02	+
BG 45	First isolate	ATM	3.68	+
BG 46	2	ATM	2.58	+
BG 47	First isolate	PIP/TAZ, ATM, TM	3.79	+
BG 48	First isolate	ATM	2.57	+
BG 49	2	ATM	2.96	+
BG 50	First isolate	ATM	2.50	+
BG 52	First isolate	ATM	1.56	+
BG 53	2	ATM	2.35	+
BG 54	First isolate	ATM	3.26	+
BG 55	3	Susceptible	6.82	+
BG 56	First isolate	ATM, AK, TM, CIP, LV	1.88	+
BG 57	First isolate	ATM, MEM, IMP	5.88	+
BG 58	2	ATM	0.70	+
BG 59	First isolate	ATM	1.18	+
BG 60	First isolate	Susceptible	2.87	+
BG 61	2	ATM	4.09	+
BG 62	First isolate	ATM	3.31	+
BG 63	2	ATM	1.87	+
BG 64	First isolate	ATM	0.01	+
BG 65	First isolate	ATM	0.00	+
BG 66	First isolate	Susceptible	2.50	+
BG 67	First isolate	Susceptible	0.00	+
BG 68	First isolate	ATM	2.63	+
BG 69	First isolate	ATM	2.57	+
BG 70	First isolate	ATM	0.01	+

BG 71	First isolate	ATM	3.44	-
BG 72	First isolate	ATM	4.04	+
BG 73	First isolate	ATM	2.52	+
BG 74	First isolate	ATM	2.88	+
BG 75	First isolate	ATM	4.37	+
BG 76	7	ATM, CAZ, IMP, AK	5.61	+
BG 77	6	PIP/TAZ, ATM, CAZ, MEM, IMP, AK, TM, CIP, LV	0.01	+
BG 78	5	CAZ, MEM, IMP, TM, CIP, LV	0.00	+
BG 79	7	ATM, TM	6.20	+
BG 80	5	AK, CIP, LV	2.85	+
BG 81	5	ATM, CIP, LV	0.01	+
BG 82	6	ATM, MEM, AK, TM, CIP, LV	2.68	+
BG 83	5	PIP/TAZ, ATM, TM	4.79	+
BG 84	5	PIP/TAZ, ATM, AK	2.00	+
BG 85	6	MEM, AK, TM	4.70	+
BG 86	6	Susceptible	3.29	+
BG 87	6	Susceptible	2.13	+
BG 88	5	CAZ, MEM, IMP, AK, TM	0.00	+
BG 89	6	MEM, IMP, AK, TM, CIP, LV	0.02	+
BG 90	7	Susceptible	2.12	+
BG 91	≥ 15	PIP/TAZ, ATM, CAZ, MEM, IMP, AK, TM, CIP, LV	2.59	+
BG 92	≥ 15	PIP/TAZ, ATM, CAZ, MEM, IMP, AK, TM, CIP, LV	0.01	+
BG 93	≥ 15	MEM, IMP, TM, CIP, LV	1.15	+
BG 95	≥ 15	ATM, CAZ, MEM, IMP, AK, TM, CIP, LV	0.02	-
BG 96	≥ 15	AK, TM	1.04	+
BG 97	≥ 15	PIP/TAZ, ATM, CAZ, MEM, IMP, AK, TM, CIP, LV	1.02	+
BG 98	≥ 15	AK, TM, CIP, LV, COL	1.91	+
BG 99	≥ 15	ATM, CAZ, MEM, IMP, AK, TM	0.00	+
BG 100	≥ 15	ATM, AK, TM, CIP, LV	1.02	+

Acronyms: PIP/TAZ, piperacillin/tazobactam; ATM, aztreonam; CAZ, ceftazidime; MEM, meropenem; IMP, imipenem; AK, amikacin; TM, tobramycin; CIP, ciprofloxacin; LV, levofloxacin; COL, colistin.

[a]PVD was determined in culture supernatants of *P. aeruginosa* strains grown for 48 h at 37°C in CAA. PVD quantities were measured as the OD_{405} of culture supernatants appropriately diluted in 0.1 M Tris-HCl, pH 8, according to[17]. Non-producers are highlighted in grey.

[b]PCH production was determined according to Supplementary text S2. +, PCH producer; -, PCH non-producer (highlighted in grey). PCH non-producers were confirmed by the microplate assay described in Supplementary text S1.

Table S5. PCH detection in sputum samples from CF patients

Sample	Culture-positive for	PCH (nM) in sputum (mean ± SD)
**1**	*P. aeruginosa* and *Staphylococcus aureus*	< 5.0
**2**	*P. aeruginosa*	86.9 ± 4.6
**3**	*P. aeruginosa*	347.6 ± 5.7
**4**	*P. aeruginosa*	230.0 ± 7.1
**5**	*P. aeruginosa*	146.1 ± 12.5
**6**	*Acinetobacter baumannii* and *S. aureus*	< 5.0
**7**	*P. aeruginosa*	108.4 ± 6.3
**8**	*P. aeruginosa*	24,390.0 ± 91.2

Supplemental text S1

Protocol for quantification of PCH in *Pseudomonas aeruginosa* culture supernatants and CF sputa by means of the *P. aeruginosa* Δ*pvdA*Δ*pchD*Δ*fpvA* P*pchE::lux* biosensor

Materials

1. Bacterial strains

1.1. *P. aeruginosa* strains are stored in LB (Luria Bertani) broth with 15 % glycerol in cryotubes at −80 °C and grown in LB plates. The *P. aeruginosa* biosensor strain Δ*pvdA*Δ*pchD*Δ*fpvA* P*pchE::lux* is freely available to the scientific community and can be provided upon request to the Corresponding Authors (Email addressed: daniela.visaggio@uniroma3.it; paolo.visca@uniroma3.it).

1.2. Preparation of the Iron-poor Casamino acids (DCAA) medium[18]

Weight 5 g of Chelex 100 resin (Bio-Rad, Hercules, CA, USA), and activate it by gentle stirring for 1 h in *ca.* 100 mL of 0.1 M HCl prepared in deionized, double-distilled water (ddH_2O). Decant and repeatedly rinse the resin with ddH_2O, until the pH of the resin suspension is 6.5. Dissolve 5 g of Casamino acids (CAA, Becton Dickinson, Franklin Lakes, NJ, USA) in *ca.* 900 mL of ddH_2O, adjust the pH to 7.2 by adding few drops of 1 M NaOH (prepared in ddH_2O), and combine it with the activated resin. After 16-h moderate stirring at 4 °C, remove the resin by filtration through Whatman no. 1 filter paper, adjust the volume to 1 L with ddH_2O, and sterilize by autoclaving. Store at room temperature. Aseptically supplement with filter-sterilized 0.4 mM $MgCl_2$ immediately prior to inoculation.

1.3. Preparation of Pyochelin (PCH) solution

A 40 mM stock solution of commercially available PCH (Santa Cruz Biotechnology Inc., Dallas, TX, USA; Cat. No. sc-506665) is prepared in dimethyl sulfoxide (Sigma-Aldrich, St. Louis, MO, USA) and stored at –20°C until used.

1.4. Preparation of ferric chloride ($FeCl_3$) solution

A stock solution of 0.1 M $FeCl_3$ (Sigma-Aldrich) in 0.1 M HCl is prepared and stored at – 20°C until used.

2. Equipment

2.1. High-speed centrifuge

2.2. UV-Visible spectrophotometer.

2.3. Plate incubator set at 25 °C.

2.4. 96-well plate reader with bioluminescence detection module (*e.g.*, Tecan Sparks 10M multilabel plate reader; Tecan, Männedorf, Switzerland).

Method (see Graphical workflow on p. xxi)

1a. Culture supernatants of test strain

1a.1. Inoculate 2-3 colonies of the *P. aeruginosa* test strain in 10 mL DCAA, using 50 mL sterile tubes. Grow bacteria for 24 h at 37 °C with shaking (180 rpm).

1a.2. Measure bacterial growth spectrophotometrically (λ = 600 nm).

1a.3. Centrifuge cultures at 10,000 x *g* for 10 min and collect the supernatant.

1a.4. Filter the supernatant through a 0.45 µm filter and store at -20 °C until use.

1b. CF sputa

1b.1. Centrifuge the sputum at 13,000 x *g* for 10 min at 4°C and collect the supernatant (5 µL).

1b.4. Dilute the sputum supernatant 10^{-1}, 10^{-2} and 10^{-3} in DCAA, then store at -20 °C until use.

2. Biosensor pre-inoculum

2.1. Inoculate 2-3 colonies of the *P. aeruginosa* Δ*pvdA*Δ*pchD*Δ*fpvA* P*pchE::lux* biosensor strain in 10 mL DCAA supplemented with 1 µM $FeCl_3$ in a 50 mL tube. Grow bacteria for 16 h at 37 °C with shaking (180 rpm).

2.2. Centrifuge the biosensor culture for 10 min at 2,500 x *g* and discard the supernatant. Wash the bacterial pellet once with saline (0.9% NaCl). Resuspend the pellet in 3 mL of DCAA and spectrophotometrically measure the concentration of the bacterial suspension (OD_{600}).

3. Biosensor calibration

3.1. Prepare a working solution of 640 nM PCH in DCAA to calibrate the biosensor in the 5-320 nM PCH concentration range.

3.2. Black, clear-bottom 96-wells microtiter plates must be used for the assay (*e.g.*, Thermo Fisher Scientific, Waltham, MA, USA; cat. no. 165305). The first row (A) of the microtiter plate is dedicated to the assay calibration. In the first well of the row (A1) dispense 50 µL of the PCH working solution, and 25 µL of DCAA in the remaining wells (A2-A12).

3.3. Perform two-fold serial dilutions using 25 µL in wells A1-A11, then discard 25 µL from well A11 (all wells should contain 25 µL). The A12 well does not contain PCH and serves as the blank for basal bioluminescent emission.

3.4. Suspend the biosensor cells in DCAA to reach OD_{600} = 0.5. Dispense 25 µL of the biosensor cell suspension in wells A1-A12 (final concentration OD_{600} = 0.25).

3.5. Incubate the 96-well plate at 25 °C for 3.5 h.

3.6. Measure both OD_{600} and bioluminescent emission (expressed as light count per second, LCPS) using a 96-well plate reader (*e.g.,* Tecan Sparks 10M multilabel plate reader; Tecan, Männedorf, Switzerland).

3.7 Subtract the blank value determined for well A12, and plot the bioluminescent emissions values normalized by OD_{600} of the biosensor (relative luminescence = LCPS/OD_{600}) as a function of PCH concentrations. Fit an equation to the data obtained, and use it to extrapolate the PCH concentration in *P. aeruginosa* supernatants (see par. 5.2).

4. *Quantification of PCH in* P. aeruginosa *culture supernatants or CF sputa*

4.1. Dilute the culture supernatants of *P. aeruginosa* test strains 1:1,000 in DCAA. Lower dilutions (1:500 or 1:250) can also be used for re-testing low PCH producers or non-producers.

4.2. Suspend the biosensor cells in DCAA to reach OD_{600} = 0.5.

4.3 Dispense 25 µL of the biosensor cell suspension in each wells of a black, clear-bottom 96-wells microtiter plate. Use rows B to H for PCH quantification in culture supernatants of test strains. Note that calibration curve and PCH quantification can be performed simultaneously in the same 96-well microplate.

4.4 Add 5 µL of either diluted *P. aeruginosa* supernatant or diluted CF sputum and 20 µL of DCAA in each well of rows B to H, in order to dilute the biosensor cells to OD_{600} = 0.25 in a final volume of 50 µL. Perform the test at least in triplicate for each diluted supernatant or CF sputum.

4.5 Incubate the 96-well plate at 25 °C for 3.5 hours.

5. Plate reading and data analysis

5.1. Measure both OD_{600} and bioluminescence emission (LCPS) using a 96-well plate reader.

5.2. Analyze the data by subtracting the blank value derived from well A12, and by plotting the bioluminescent emission values normalized by OD_{600} of the biosensor (relative luminescence) onto the calibration curve obtained as described (par. 3.7). The resulting PCH concentration must be multiplied by 10x the supernatant dilution factor (*i.e.*, 10,000, 5,000 or 2,500, depending on sample dilution; see par. 4.1).

Supplemental text S2

Protocol for the detection of PCH production on solid agar plates by means of the *P. aeruginosa* Δ*pvdA*Δ*pchD*Δ*fpvA* P*pchE::lux* biosensor

Materials

1. Bacterial strains

1.1. *P. aeruginosa* strains are stored in LB (Luria Bertani) broth with 15 % glycerol in cryotubes at −80 °C and grown in LB plates. The *P. aeruginosa* biosensor strain Δ*pvdA*Δ*pchD*Δ*fpvA* P*pchE::lux* is freely available to the scientific community and can be provided upon request to the Corresponding Authors (Email addresses: daniela.visaggio@uniroma3.it; paolo.visca@uniroma3.it).

1.2. Preparation of the iron-poor Casamino acids (DCAA) medium[18]

Weight 5 g of Chelex 100 resin (Bio-Rad, Hercules, CA, USA) and activate it by gentle stirring for 1 h in *ca.* 100 mL of 0.1 M HCl prepared in deionized, double-distilled water (ddH_2O). Decant and repeatedly rinse the resin with ddH2O, until the pH of the resin suspension is 6.5. Dissolve 5 g of Casamino acids (CAA, Becton Dickinson, Franklin Lakes, NJ, USA) in *ca.* 900 mL of ddH_2O, adjust the pH to 7.2 by adding few drops of 1 M NaOH (prepared in ddH_2O), and combine it with the activated resin. After 16 h of moderate stirring at 4 °C, remove the resin by filtration through Whatman no. 1 filter paper, adjust the volume to 1 L with ddH2O, and sterilize by autoclaving. Store at room temperature. Aseptically supplement with filter-sterilized 0.4 mM $MgCl_2$, immediately prior to inoculation.

1.3. Preparation of CAA plates

Dissolve 10 g of CAA in 1 l of ddH_2O (2X CAA) and sterilize by autoclaving. Store the medium at room temperature. Prepare 3% (w/v) agarose in ddH_2O using a Certified Molecular Biology Agarose (Bio-Rad). Incubate 2X CAA and molten 3% agarose at 45°C and 55 °C, respectively, then mix them in 1:1 ratio before pouring plates. For the preparation of the agarose top layer, dissolve 16 g/L (1.6 % w/v) Certified Molecular Biology Agarose (Bio-Rad) in sterile ddH_2O.

1.4. Preparation of ferric chloride ($FeCl_3$) solution

A stock solution of 0.1 M $FeCl_3$ (Sigma-Aldrich) in 0.1 M HCl is prepared and stored at – 20°C until used.

2. Equipment

2.1. Chemical hood

2.2. UV-Visible spectrophotometer.

2.3. Incubator set at 37 °C.

2.4. Bioluminescence imaging system (*e.g.*, Bio-Rad ChemiDoc XRS + Imaging System; Bio-Rad)

Methods (see Graphical workflow on p. xxi)

1. Spot-inoculation of test strains on CAA plates

1.1. Inoculate 2-3 colonies of the *P. aeruginosa* test strains in 3 mL LB, using 15-mL sterile tubes. Grow bacteria for 8 h at 37 °C with shaking (180 rpm).

1.2. Measure bacterial growth spectrophotometrically (λ = 600 nm) and adjust to $OD_{600} \cong 0.1$ in LB.

1.3. Inoculate 5 µL of the diluted bacterial cultures onto the surface of CAA plates to obtain a spot ≅ 2 mm. Incubate the plate for 24 h at 37 °C.

2. Chloroform treatment

2.1. Working under a chemical hood, impregnate a filter paper disk (*ca.* 8 cm diameter) with chloroform (*ca.* 2 mL) and place the CAA plate upside-down over the disk for 15 min to allow the chloroform vapor kill bacteria.

2.2. Expose the plate to air for additional 15 min to evaporate residual chloroform.

3. *Biosensor pre-inoculum and treatment*

3.1. Inoculate 2-3 colonies of the *P. aeruginosa* Δ*pvdA*Δ*pchD*Δ*fpvA* P*pchE::lux* biosensor strain in 10 mL DCAA supplemented with 1 µM $FeCl_3$ in a 50 mL flask. Grow bacteria for 16 h at 37 °C with shaking (180 rpm).

3.2. Centrifuge the biosensor culture for 10 minutes at 2,500 x *g* and discard the supernatant. Wash the bacterial pellet once with saline (0.9% NaCl). Suspend the pellet in 3 mL of DCAA and spectrophotometrically measure the concentration of the bacterial suspension (OD_{600}).

4. Biosensor overlay

4.1. Suspend the biosensor cells in 2X CAA to reach $OD_{600} = 0.5$.

4.2. Prepare the overlay solution by mixing the diluted biosensor suspension with molten 1.6% agarose (pre-heated and kept at 45 °C to prevent jelling) in a 1:1 ratio.

4.3. Pre-warm the chloroform-treated CAA plates at 42°C and overlay the biosensor cell suspension on it.

5. Plate reading

5.1. Incubate the CAA plate at 25 °C for 1 hour.

5.2. Visualize the CAA plate with a bioluminescence imaging system and appropriate exposition settings. If using a Bio-Rad ChemiDoc XRS + Imaging System, the Chemi Hi Resolution application must be set with no filter, no illumination and 3 min exposure time.

Graphical workflow. Schematic illustration of the steps required for PCH detection on solid medium and PCH quantification from liquid samples.

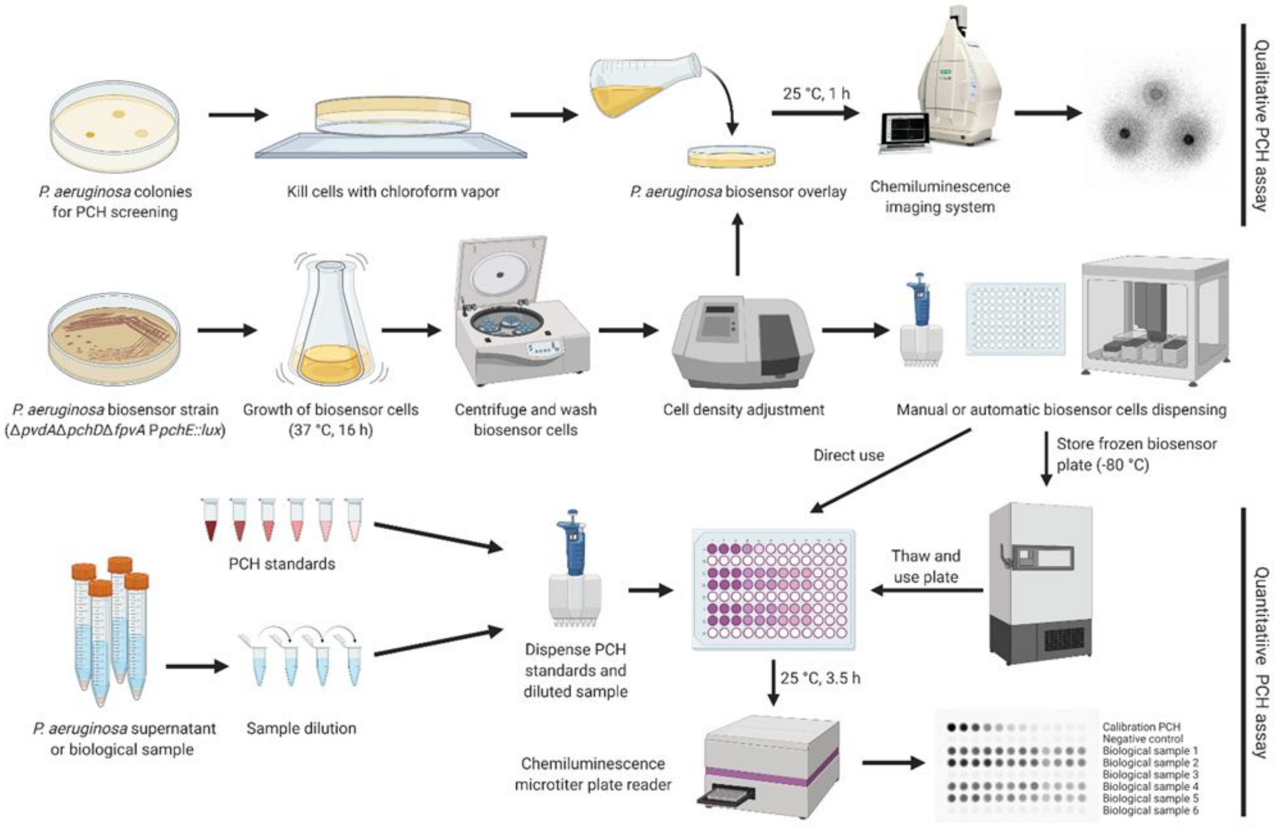

References

(1) Sambrook, J.; Fritsch, E. F.; Maniatis, T. Molecular Cloning: A Laboratory Manual. *Molecular cloning: a laboratory manual.* **1989**, No. Ed. 2.

(2) Simon, R.; Priefer, U.; Pühler, A. A Broad Host Range Mobilization System for In Vivo Genetic Engineering: Transposon Mutagenesis in Gram Negative Bacteria. *Nat. Biotechnol.* **1983**, *1*, 784–791.

(3) Holloway, B. W. Genetic Recombination in *Pseudomonas aeruginosa*. *J. Gen. Microbiol.* **1955**, *13*, 572–581.

(4) Imperi, F.; Putignani, L.; Tiburzi, F.; Ambrosi, C.; Cipollone, R.; Ascenzi, P.; Visca, P. Membrane-Association Determinants of the Omega-Amino Acid Monooxygenase PvdA, a Pyoverdine Biosynthetic Enzyme from *Pseudomonas aeruginosa*. *Microbiology (Reading)* **2008**, *154*, 2804–2813.

(5) Frangipani, E.; Bonchi, C.; Minandri, F.; Imperi, F.; Visca, P. Pyochelin Potentiates the Inhibitory Activity of Gallium on *Pseudomonas aeruginosa*. *Antimicrob. Agents Chemother.* **2014**, *58*, 5572–5575.

(6) Visca, P.; Bonchi, C.; Minandri, F.; Frangipani, E.; Imperi, F. The Dual Personality of Iron Chelators: Growth Inhibitors or Promoters? *Antimicrob. Agents Chemother.* **2013**, *57*, 2432–2433.

(7) Medeiros, A. A.; O'Brien, T. F.; Wacker, W. E. C.; Yulug, N. F. Effect of Salt Concentration on the Apparent In-Vitro Susceptibility of *Pseudomonas* and Other Gram-Negative Bacilli to Gentamicin. *J. Infect. Dis*. **1971**, *124*, S59–S64.

(8) Rahme, L. G.; Stevens, E. J.; Wolfort, S. F.; Shao, J.; Tompkins, R. G.; Ausubel, F. M. Common Virulence Factors for Bacterial Pathogenicity in Plants and Animals. *Science* **1995**, *268*, 1899–1902.

(9) Cheng, K.; Smyth, R. L.; Govan, J. R.; Doherty, C.; Winstanley,C.; Denning, N.; Heaf, D. P.; van Saene, H.; Hart, C. A. Spread of β-Lactam-Resistant *Pseudomonas aeruginosa* in a Cystic Fibrosis Clinic. *Lancet* **1996**, *348*, 639–642.

(10) Massai, F.; Imperi, F.; Quattrucci, S.; Zennaro, E.; Visca, P.; Leoni, L. A Multitask Biosensor for Micro-Volumetric Detection of N-3-Oxo-Dodecanoyl-Homoserine Lactone Quorum Sensing Signal. *Biosens. Bioelectron.* **2011**, *26*, 3444–3449.

(11) Bragonzi, A.; Paroni, M.; Nonis, A.; Cramer, N.; Montanari, S.; Rejman, J.; Di Serio, C.; Döring, G.; Tümmler, B. *Pseudomonas aeruginosa* Microevolution during Cystic Fibrosis Lung Infection Establishes Clones with Adapted Virulence. *Am. J. Respir. Crit. Care Med.* **2009**, *180*, 138–145.

(12) Bonchi, C.; Frangipani, E.; Imperi, F.; Visca, P. Pyoverdine and Proteases Affect the Response of *Pseudomonas aeruginosa* to Gallium in Human Serum *Antimicrob. Agents Chemother*. **2015**, *59*, 5641–5646.

(13) Milton, D. L.; O'Toole, R.; Horstedt, P.; Wolf-Watz, H. Flagellin A Is Essential for the Virulence of *Vibrio anguillarum*. *J. Bacteriol.* **1996**, *178*, 1310–1319.

(14) Voisard, C.; Bull, C.; Keel, C.; Laville, J.; Maurhofer, M.;Schnider, U.; Défago, G.; Haas, D. In O'Gara, F.; Dowling, D.N.; Boesten, B. (Eds) Molecular Ecology of Rhizosphere Microorganisms **1994**, 67-89.

(15) Hoang, T. T.; Kutchma, A. J.; Becher, A.; Schweizer, H. P. Integration-Proficient Plasmids for *Pseudomonas aeruginosa*: Site-Specific Integration and Use for Engineering of Reporter and Expression Strains. *Plasmid* **2000**, *43*, 59–72.

(16) Hoang, T. T.; Karkhoff-Schweizer, R. R.; Kutchma, A. J.; Schweizer, H. P. A Broad-Host-Range Flp-FRT Recombination System for Site-Specific Excision of Chromosomally-Located DNA Sequences: Application for Isolation of Unmarked *Pseudomonas aeruginosa* Mutants. *Gene* **1998**, *212*, 77–86.

(17) Visca, P.; Serino, L.; Orsi, N. Isolation and Characterization of *Pseudomonas aeruginosa* Mutants Blocked in the Synthesis of Pyoverdin. *J. Bacteriol*. **1992**, *174*, 5727–5731.

(18) Visca, P.; Colotti, G.; Serino, L.; Verzili, D.; Orsi, N.; Chiancone, E. Metal Regulation of Siderophore Synthesis in *Pseudomonas aeruginosa* and Functional Effects of Siderophore-Metal Complexes. *Appl. Environ. Microbiol*. **1992**, *58*, 2886–2893.
